# A methodological framework for integrating generalisable deep learning and radiomics fusion model for early lung cancer detection across multi-centre imaging datasets

**DOI:** 10.1186/s12911-026-03624-9

**Published:** 2026-06-11

**Authors:** Ndivho Tshivhula, Elliot Mbunge, Tebogo Makaba

**Affiliations:** 1 Center for Applied Data Science, University of Johannesburg, Johannesburg, 2006 South Africa; 2 Department of Applied Information Systems, University of Johannesburg, Johannesburg, South Africa

**Keywords:** Lung cancer, Deep learning, Radiomics, Fusion model, CT imaging, PET CT, Clinical decision support system, Explainable AI, Generalisability

## Abstract

Early detection of lung cancer remains one of the most effective strategies for improving survival; however, diagnostic performance is limited by variability in imaging protocols, scanner settings, lesion characteristics, and inter-reader interpretation. Deep learning (DL) models enable automated feature representation from medical images, while radiomics provides interpretable handcrafted biomarkers that capture tumour morphology, intensity, texture, and heterogeneity. When integrated into a clinical decision support system (CDSS), these approaches have the potential to augment radiologist decision-making, reduce false positives, and improve triage of indeterminate pulmonary nodules. However, both approaches continue to face challenges related to reproducibility, explainability, and generalisability across multi-centre imaging datasets. This systematic review followed PRISMA 2020 guidelines and synthesised evidence on CT-, LDCT-, spectral CT-, and PET/CT-based radiomics, DL, and DL–radiomics fusion models for early lung cancer detection and classification. Following revised searches across Scopus, PubMed/MEDLINE, IEEE Xplore, and Google Scholar, and subsequent eligibility screening, the retained core detection and classification studies were organised into three analytical categories: radiomics-only models, DL-only models, and DL–radiomics fusion models. Reported performance metrics, validation strategies, imaging modalities, fusion approaches, and centre designs were compared to identify methodological patterns and sources of heterogeneity. Fusion models generally outperformed radiomics-only and DL-only approaches across benign–malignant nodule classification, lung cancer classification, invasive adenocarcinoma prediction, ground-glass nodule assessment, and histological subtyping. Late and hybrid fusion strategies were more robust than early feature concatenation, particularly when handcrafted radiomics, deep features, and clinical variables (e.g., smoking history, spiculation, tumour stage, PET/CT indicators) were combined. However, the evidence base is constrained by retrospective designs, small samples, inconsistent segmentation and harmonisation, limited prospective validation, and incomplete reporting of calibration, decision-curve analysis, and external performance. Formal statistical pooling was not appropriate given the heterogeneity in outcomes, imaging protocols, feature pipelines, model architectures, and validation strategies. To address these gaps, this review proposes a methodological framework integrating multi-centre harmonisation, robust feature engineering, mathematically defined fusion strategies, domain generalisation, explainability, calibration, and clinical utility assessment to support reproducible and clinically meaningful CDSS for early lung cancer detection.

## Clinical Trial Number

Not applicable.

## Introduction

Lung cancer remains the leading cause of cancer-related mortality globally, with survival outcomes highly dependent on early diagnosis [1, 2]. Low-dose computed tomography (LDCT) screening has demonstrated significant reductions in lung cancer mortality in major trials such as the National Lung Screening Trial (NLST) and the NELSON trial [3, 4]. However, the effectiveness of LDCT screening is constrained by high image-reading workload, false-positive findings, interpretative variability, and the need for reliable tools to support early detection and risk stratification. Clinical decision support systems (CDSS) built on artificial intelligence have therefore emerged as a promising mechanism for augmenting radiologist interpretation, prioritising suspicious findings, and standardising decision-making across centres.

Deep learning (DL) approaches have shown promising results in automated pulmonary nodule detection, lesion classification, and image-based lung cancer diagnosis. These models can learn complex hierarchical representations directly from CT or LDCT images. In parallel, radiomics provides interpretable quantitative biomarkers that describe tumour morphology, intensity, texture, shape, and heterogeneity [5, 6]. Despite these advantages, both approaches face important barriers to clinical translation. DL models are often criticised for limited transparency and vulnerability to dataset bias, while radiomics pipelines are affected by variability in image acquisition, segmentation, preprocessing, feature extraction, and reconstruction protocols [7]. For CDSS deployment, these limitations are particularly important because clinically deployed models must be reproducible, interpretable, and robust across diverse imaging environments.

A major limitation across both DL and radiomics studies is poor generalisability. Many models are developed using single-centre retrospective datasets and may experience performance degradation when applied to external cohorts, different scanners, alternative reconstruction kernels, or demographically diverse populations [8, 9]. Limited external validation, inconsistent feature harmonisation, and incomplete explainability reporting restrict the clinical adoption of these models, particularly in multi-centre imaging settings where robust performance across institutions is essential for safe and scalable CDSS deployment [7, 10].

DL–radiomics fusion models have emerged as a promising strategy to address some of these limitations. These models combine the automated representation-learning capacity of DL with the interpretability of handcrafted radiomic descriptors. Such hybrid approaches can integrate CNN- or transformer-derived deep features with radiomics, clinical variables, semantic radiological features, or PET/CT-derived parameters, thereby capturing complementary information from multiple feature spaces [11]. However, fusion studies vary substantially in their imaging modalities, outcome definitions, feature extraction pipelines, fusion strategies, validation designs, and reporting quality. In addition, explainability frameworks such as Grad-CAM and SHAP remain inconsistently integrated into clinical workflows [12, 13], which limits the trust and adoption of such models within CDSS pipelines.

There is therefore a critical need to systematically synthesise the current evidence on radiomics-only, DL-only, and DL–radiomics fusion models for early lung cancer detection and classification. Such a synthesis is necessary to clarify methodological patterns, identify sources of heterogeneity, assess the role of external validation and explainability, and inform the design of future generalisable fusion models intended for clinical decision support. Accordingly, this study aims to evaluate and synthesise current evidence on DL–radiomics fusion models for early lung cancer detection and classification, with particular emphasis on multi-centre generalisability, fusion strategy, validation design, explainability, calibration, and clinical utility, and to propose a methodological framework to guide their development as CDSS components.

To achieve this aim, the study addressed the following research questions:

- Which radiomics-only, DL-only, and DL–radiomics fusion models have been used for early lung cancer detection and classification?
- Which methodological approaches improve generalisability across multi-centre imaging datasets and support explainability integration?
- How are radiomics features, deep learning features, and clinical or semantic variables integrated across early, late, and hybrid fusion strategies?
- What proposed methodological framework can guide the design, validation, and clinical integration of generalisable DL–radiomics fusion models as CDSS for early lung cancer detection?

The remainder of this paper is structured as follows. Section “Materials and methods” presents the materials and methods, including the PRISMA 2020-guided search strategy, eligibility criteria, study selection, data extraction, and synthesis approach. Section “Results and discussion” presents the results and discussion, including the structured synthesis of radiomics-only, DL-only, and DL–radiomics fusion models, the taxonomy of fusion strategies, sources of heterogeneity, and methodological implications. Section “Proposed methodological framework for generalisable DL–radiomics fusion as a clinical decision support system” presents the proposed methodological framework for generalisable DL–radiomics fusion models as CDSS. Section “Limitations of the study and future work” presents the limitations of the study and future work. Section “Conclusion” concludes the paper.

## Materials and methods

**Fig. 1:**
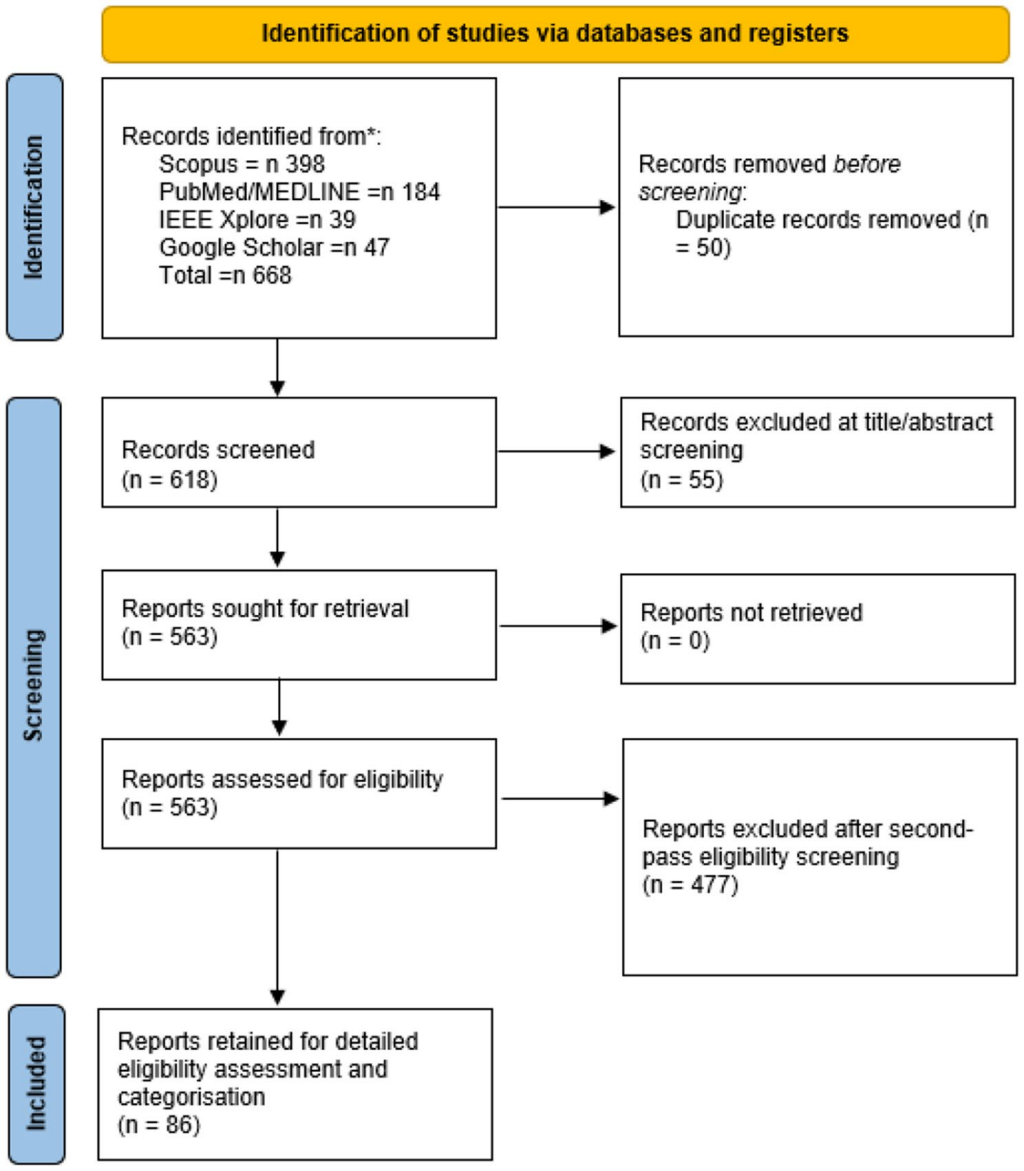
PRISMA flow diagram showing identification, screening, duplicate removal, second-pass eligibility screening, and 86 records included in the structured quantitative synthesis

This systematic review was conducted and reported in accordance with the Preferred Reporting Items for Systematic Reviews and Meta-Analyses (PRISMA 2020) guidelines [14]. PRISMA 2020 provides a structured and transparent framework for reporting literature identification, screening, eligibility assessment, and synthesis. In line with these recommendations, a PRISMA 2020 flow diagram is presented in Fig. 1, detailing the number of records identified, screened, excluded, and retained for inclusion in the structured quantitative synthesis. This approach was used to enhance methodological transparency, reproducibility, and traceability across the revised search and screening process.

### Search strategy

The revised database search retrieved 398 records from Scopus, 184 records from PubMed/MEDLINE, and 39 records from IEEE Xplore. In addition, 47 Google Scholar records were screened as a supplementary source, resulting in 668 records before duplicate removal.

The search strategy combined controlled vocabulary and free-text terms relating to lung cancer, pulmonary nodules, computed tomography, low-dose CT, PET/CT, radiomics, deep learning, artificial intelligence, and model fusion. Boolean operators were used to maximise sensitivity while maintaining relevance to the review objective. The search strategy was adapted for each database according to its indexing requirements. For Scopus, the search was implemented using TITLE-ABS-KEY; for PubMed/MEDLINE, Medical Subject Headings (MeSH) and Title/Abstract field tags were used; and for IEEE Xplore and Google Scholar, equivalent free-text combinations were applied. Searches were restricted to English-language studies published between January 2014 and December 2025.

The Scopus search string was:

**Figure:**
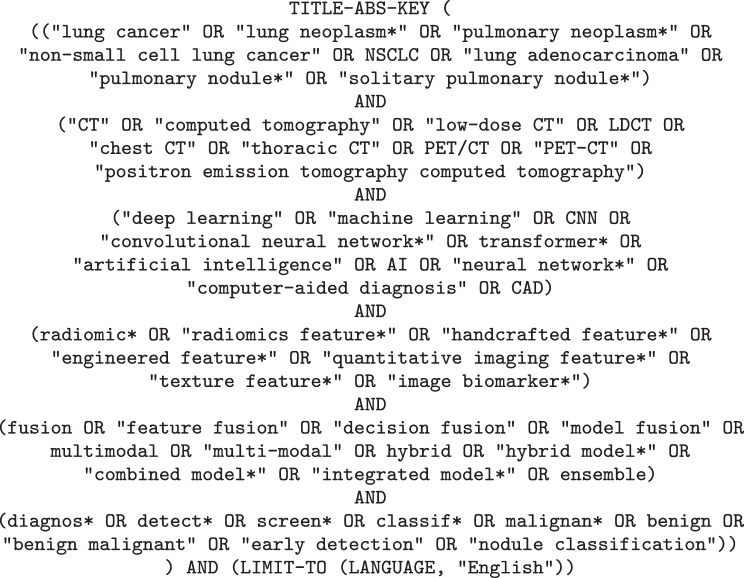

The PubMed/MEDLINE search string was:

**Figure:**
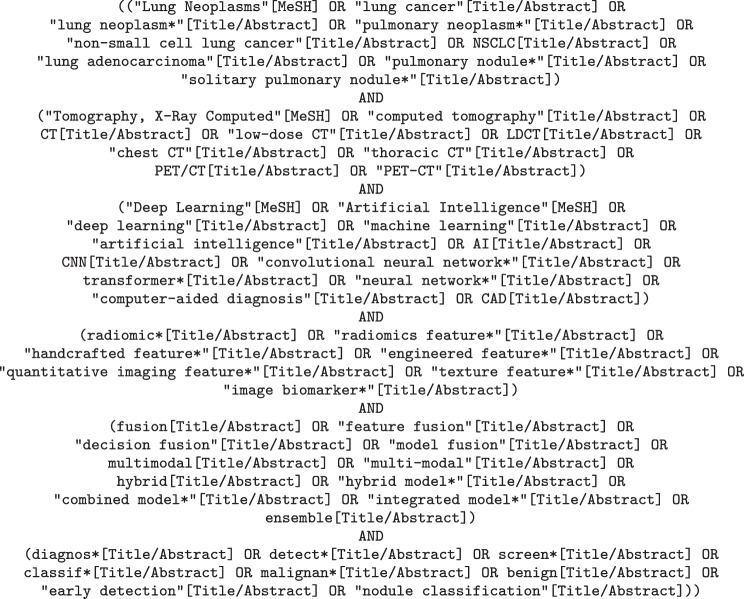

The IEEE Xplore search string was adapted using equivalent free-text terms as follows:

**Figure:**
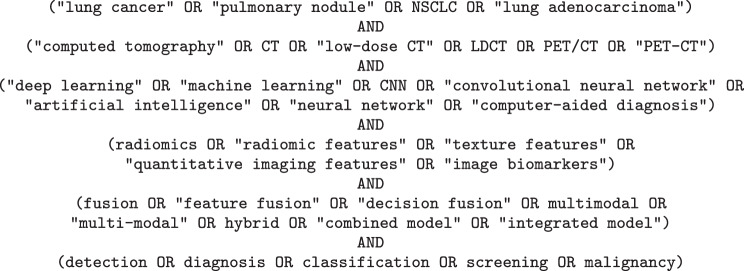

For Google Scholar, shorter keyword combinations were used because of search-length limitations and the large number of non-specific results returned by the platform. The predefined Google Scholar keyword combination was:

**Figure:**
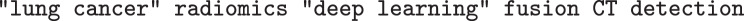

Due to the ranking-based retrieval structure and high duplication rate in Google Scholar, the first 47 relevant Google Scholar records were manually screened by title and snippet as a supplementary source. Potentially relevant records were entered into the screening spreadsheet and assessed against the same eligibility criteria applied to the database searches.

The revised search retrieved 398 records from Scopus, 184 records from PubMed/MEDLINE, and 39 records from IEEE Xplore. In addition, 47 Google Scholar records were screened as a supplementary source, resulting in 668 records before duplicate removal. The search strategy was designed to identify studies focusing on radiomics, deep learning, and DL–radiomics fusion models applied to CT, LDCT, spectral CT, or PET/CT imaging for lung cancer detection, diagnosis, screening, pulmonary nodule classification, histological subtype classification, and invasiveness prediction.

Duplicate records were removed before title-and-abstract screening. Manual citation snowballing was also performed by reviewing the reference lists of eligible studies and relevant review articles to identify additional potentially eligible publications not captured through database searching. All records identified through database searching, Google Scholar screening, and citation snowballing were assessed against the predefined eligibility criteria before inclusion in the final synthesis.

### Protocol registration and transparency

This systematic review was not prospectively registered in PROSPERO or another public protocol registry. However, the review question, eligibility criteria, search strategy, data-extraction variables, and synthesis plan were defined before full-text screening and are reported transparently in the Methods Section “Proposed Methodological Framework for Generalisable DL–Radiomics Fusion as a Clinical Decision Support System” to support reproducibility. The absence of prospective registration is acknowledged as a methodological limitation. To minimise reporting bias and improve transparency, the review was conducted and reported in accordance with PRISMA 2020 guidance [14].

### Inclusion and exclusion criteria

Eligibility criteria were defined a priori using the Population, Intervention, Comparator, Outcomes, and Study design (PICOS) framework. The PICOS framework is widely used in evidence-based medicine to structure focused research questions and to ensure that study selection is transparent and aligned with the primary clinical question. It specifies the target population (P), intervention or exposure (I), comparator (C), outcomes of interest (O), and study designs to be included or excluded [15].

In this review, the Population referred to human participants undergoing lung cancer screening, diagnosis, or pulmonary nodule assessment using CT, LDCT, spectral CT, or PET/CT imaging. The Intervention was defined as AI-based imaging models, including radiomics-only models, deep learning-only models, and DL–radiomics fusion models. Fusion models were defined as approaches integrating deep learning-derived image features with handcrafted radiomic features and, where applicable, clinical, semantic radiological, or PET/CT-derived variables.

Comparators included radiomics-only models, DL-only models, fusion models, conventional machine-learning models, or clinical/imaging-based diagnostic approaches. Outcomes included quantitative diagnostic or classification performance metrics such as AUC, accuracy, sensitivity, specificity, precision, F1-score, false-positive reduction, calibration, external validation performance, and measures of explainability or robustness where reported. Core outcomes included lung cancer detection, benign–malignant pulmonary nodule classification, lung lesion classification, histological subtype classification, malignancy prediction, and invasiveness prediction.

The Study design component focused on original empirical studies, including retrospective or prospective studies, single-centre or multi-centre studies, peer-reviewed journal articles, conference papers, and high-quality preprints published in English between January 2014 and December 2025. Only studies that satisfied the core eligibility criteria were included in the structured quantitative synthesis. Studies focused primarily on prognosis, recurrence, survival, treatment response, toxicity, EGFR/PD-L1 prediction, lymph-node metastasis, STAS, or radiogenomics were not eligible for inclusion in this review, since the review scope was restricted to early detection and classification.

These conceptual PICOS elements were operationalised into concrete inclusion and exclusion criteria used for title, abstract, and full-text screening. Only studies that satisfied the core eligibility criteria were included in the main structured quantitative synthesis, while non-empirical reports, review articles, non-CT-based imaging studies, animal-only or phantom-only studies, and papers lacking quantitative performance metrics or sufficient methodological detail were excluded. The inclusion and exclusion criteria are shown in Table 1.

**Table 1:** Inclusion and exclusion criteria

Criterion	Inclusion	Exclusion
Study focus	Studies on lung cancer detection, diagnosis, screening, pulmonary nodule classification, malignancy prediction, histological subtype classification, or invasiveness prediction using CT, LDCT, spectral CT, or PET/CT imaging.	Studies not related to lung cancer, not focused on detection/classification, or not using CT, LDCT, spectral CT, or PET/CT imaging.
Imaging modality	CT, LDCT, spectral CT, or PET/CT imaging.	MRI-only, PET-only without CT, X-ray, ultrasound, pathology-only, genomics-only, or other non-CT-based imaging studies.
Model intervention	Radiomics-only models, deep learning-only models, or DL–radiomics fusion models applied to lung cancer or pulmonary nodule detection/classification.	Studies without radiomics, deep learning, artificial intelligence-based image analysis, or relevant fusion/modelling components.
Comparator / baseline	Studies comparing fusion models with radiomics-only, DL-only, conventional machine-learning, clinical, or imaging-based baselines, where reported.	Studies with no model comparison, no baseline description, or insufficient methodological detail to interpret the model contribution.
Publication type	Peer-reviewed journal articles, conference papers, book chapters, and high-quality preprints with sufficient methodological and performance information.	Reviews, systematic reviews, editorials, letters, commentaries, theses, reports, opinion pieces, protocols, or non-empirical articles.
Year of publication	Published between January 2014 and December 2025.	Published before January 2014 or after December 2025.
Language	Written in English.	Non-English publications without an English version.
Population	Human participants or human imaging datasets used for lung cancer screening, diagnosis, pulmonary nodule assessment, or early-stage lung cancer classification.	Animal-only, phantom-only, simulation-only, or purely synthetic datasets without human imaging data.
Outcomes & reporting	Quantitative diagnostic or classification metrics reported, including AUC, accuracy, sensitivity, specificity, precision, F1-score, false-positive reduction, calibration, or external validation performance.	No experimental validation, no quantitative performance metrics, or insufficient reporting of model outputs and validation results.
Study outcome scope	Studies addressing lung cancer detection, benign–malignant nodule classification, lung lesion classification, histological subtype classification, malignancy prediction, or invasiveness prediction.	Studies on prognosis, survival, treatment response, EGFR/PD-L1 prediction, lymph-node metastasis, STAS, recurrence, radiogenomics, or other non-detection outcomes.
Relevance to review topic	Title, abstract, and full text aligned with radiomics, DL, or DL–radiomics fusion for early lung cancer detection and classification.	Irrelevant titles/abstracts, non-lung cancer applications, non-imaging studies, or methods unrelated to the review topic.

### Risk of bias assessment

The methodological quality and risk of bias of the included diagnostic and classification studies were assessed using adapted criteria from the Quality Assessment of Diagnostic Accuracy Studies 2 (QUADAS-2) tool [16]. QUADAS-2 is a validated framework designed for systematic reviews of diagnostic accuracy studies and evaluates both risk of bias and applicability concerns across four domains: patient selection, index test, reference standard, and flow and timing [16].

For this review, QUADAS-2 was adapted to the context of AI-based lung cancer detection and classification. The assessment followed the recommended QUADAS-2 process of clarifying the review question, adapting signalling questions to the review topic, constructing a study flow diagram, and judging risk of bias and applicability [16]. Review-specific signalling questions were developed to capture common sources of bias in radiomics-only, DL-only, and DL–radiomics fusion studies.

Each study was evaluated across the four QUADAS-2 domains as follows:

- **Patient selection:** This domain assessed whether participants were a consecutive or random sample of eligible patients undergoing CT, LDCT, spectral CT, or PET/CT for lung cancer screening, diagnosis, pulmonary nodule assessment, or early-stage classification; whether case-control designs were avoided; and whether inappropriate exclusions, such as indeterminate nodules or poor-quality scans, were minimised. This domain captured potential sampling and spectrum bias.
- **Index test:** This domain evaluated whether the AI model, including radiomics-only, DL-only, or DL–radiomics fusion models, was developed and interpreted without knowledge of the reference standard; whether decision thresholds were pre-specified; and whether model development, feature selection, and validation were clearly separated to reduce the risk of overfitting or information leakage.
- **Reference standard:** This domain examined whether the reference standard used to define lung cancer status, nodule malignancy, histological subtype, or invasiveness was likely to correctly classify the target condition, and whether reference standard interpretation was independent of the index test results.
- **Flow and timing:** This domain assessed whether all participants received the same or an appropriate reference standard; whether the interval between imaging, index test, and reference standard was clinically appropriate; and whether all enrolled participants were included in the final analysis. This domain captured potential partial verification bias, loss to follow-up, and inappropriate exclusions.

In line with QUADAS-2 guidance, each domain was rated as low, high, or unclear risk of bias. Applicability concerns were also judged for the patient selection, index test, and reference standard domains. Two reviewers independently applied the adapted QUADAS-2 checklist to the included studies. For each judgement, supporting information from the article was recorded to improve transparency.

Discrepancies between reviewers were resolved through discussion; where consensus could not be reached, a third senior reviewer was consulted. Studies were then qualitatively categorised as follows:

- **Low overall risk of bias:** all domains were rated low risk.
- **Moderate risk of bias:** one or more domains were rated unclear, but none were rated high.
- **High risk of bias:** at least one domain was rated high risk.

Following QUADAS-2 recommendations, no numerical quality score was calculated [16]. Instead, domain-level ratings were summarised descriptively and used to interpret heterogeneity in reported diagnostic performance, validation design, and model generalisability, rather than to exclude studies post hoc.

### Data extraction and analysis

Data for potentially eligible studies published between 2014 and 2025 were extracted using a standardised data-extraction template. For each study, the following information was recorded: author(s), year of publication, country or region of origin, dataset characteristics, number of patients, number of centres, imaging modality, imaging protocol, segmentation approach, study design, model type, validation strategy, and reported performance metrics. Model-related variables included the radiomics pipeline, deep learning architecture, feature-selection method, fusion strategy, classifier, harmonisation procedure, calibration assessment, and explainability method where reported.

The revised synthesis was structured to enable comparison across the main modelling paradigms identified in the literature. Included studies were grouped into three analytical categories: radiomics-only models, deep learning-only models, and DL–radiomics fusion models. Within each category, reported area under the receiver operating characteristic curve (AUC), accuracy, sensitivity, specificity, validation strategy, dataset size, imaging modality, and centre design were extracted and compared. This categorisation enabled assessment of whether fusion models reported improved performance relative to single-stream radiomics or deep learning approaches.

A formal pooled meta-analysis and meta-regression were considered. However, statistical pooling was not performed because the included studies showed substantial methodological and clinical heterogeneity. Key sources of heterogeneity included differences in imaging modality, outcome definition, segmentation method, radiomics feature extraction pipeline, deep learning architecture, fusion strategy, classifier selection, validation design, and reporting of performance metrics. Imaging modalities included CT, LDCT, spectral CT, and PET/CT, while outcomes included lung cancer detection, benign–malignant pulmonary nodule classification, ground-glass nodule invasiveness prediction, histological subtype classification, malignancy grading, and false-positive reduction. In addition, many studies did not report confidence intervals, standard errors, confusion matrices, calibration metrics, or sufficient variance estimates required for robust pooling of diagnostic accuracy outcomes. Therefore, a formal random-effects meta-analysis was considered inappropriate and potentially misleading.

Instead, a structured quantitative synthesis and subgroup comparison were performed. Studies were stratified according to modelling paradigm, imaging modality, fusion strategy, and validation design. Modelling paradigms included radiomics-only, deep learning-only, and DL–radiomics fusion models. Imaging modality subgroups included CT/LDCT, spectral CT, and PET/CT. Fusion strategies were categorised as early, late, or hybrid/intermediate fusion where sufficient information was available. Validation designs were grouped as internal validation only, cross-validation, independent test-set validation, external validation, or multi-centre validation. Patterns in reported performance were then interpreted alongside methodological quality, validation design, reporting completeness, and risk-of-bias considerations.

The findings from this structured synthesis were used to inform the proposed methodological framework by identifying recurring methodological strengths, limitations, and design requirements for generalisable DL–radiomics fusion models in early lung cancer detection. To further support the structured synthesis, potential sources of heterogeneity were identified a priori and considered during subgroup comparison. These sources were used to interpret differences in reported model performance across radiomics-only, deep learning-only, and DL–radiomics fusion studies. Table 2 summarises the main methodological and clinical sources of heterogeneity considered in this review.

**Table 2:** Key sources of heterogeneity considered during structured quantitative synthesis

Source of heterogeneity	Potential influence on reported model performance
Imaging modality	Differences between CT, LDCT, spectral CT, and PET/CT may influence feature quality, lesion visibility, metabolic information, and model discrimination.
Dataset design	Single-centre studies may overestimate performance compared with external, multi-centre, or site-held-out validation cohorts.
Sample size and class balance	Small datasets and imbalanced benign/malignant or subtype classes may increase overfitting and inflate reported accuracy, sensitivity, specificity, or AUC.
Outcome definition	Included studies varied across benign–malignant nodule classification, lung cancer detection, subtype classification, invasiveness prediction, malignancy grading, and false-positive reduction.
Segmentation approach	Manual, semi-automatic, and automatic segmentation methods may affect radiomics reproducibility and deep feature extraction consistency.
Radiomics pipeline	Differences in preprocessing, voxel resampling, intensity normalisation, feature extraction software, feature selection, and harmonisation procedures may affect feature stability and reproducibility.
Deep learning architecture	CNNs, ResNets, DenseNets, Vision Transformers, capsule networks, and hybrid networks differ in feature representation capacity, data requirements, and generalisation behaviour.
Fusion strategy	Early, late, and hybrid fusion combine radiomics, deep learning, and clinical features at different modelling stages, making direct comparison difficult.
Validation design	Internal train–test splits and cross-validation may report higher performance than external validation, multi-centre validation, or leave-one-site-out testing.
Performance reporting	Inconsistent reporting of AUC, accuracy, sensitivity, specificity, confidence intervals, calibration, and decision-curve analysis limits statistical pooling and comparison.
Clinical and demographic variation	Differences in patient population, smoking history, tumour stage, histological subtype, scanner type, and acquisition protocol may affect model transportability.
Explainability and calibration	Limited reporting of explainability, calibration, and clinical utility makes it difficult to assess whether high-performing models are clinically reliable.

These heterogeneity sources were used to guide subgroup interpretation rather than formal statistical meta-regression, because the included studies did not consistently report comparable outcome definitions, confidence intervals, standard errors, or sufficient variance estimates required for robust pooled modelling.

### Study quality evaluation

Study quality was appraised using a bespoke checklist adapted from the Radiomics Quality Score (RQS) [17] and the Checklist for Artificial Intelligence in Medical Imaging (CLAIM) reporting guideline [18]. These tools were selected because the included studies involved radiomics feature extraction, artificial intelligence model development, validation, and reporting of diagnostic or classification performance.

The quality assessment focused on domains relevant to radiomics-only, DL-only, and DL–radiomics fusion studies. These domains included cohort size and data diversity, clarity of eligibility criteria, imaging protocol description, segmentation method, radiomics feature robustness, feature-selection procedure, model architecture transparency, validation design, use of external or multi-centre validation, calibration reporting, explainability, and availability of code, parameters, or implementation details where reported.

Studies were judged to be more methodologically robust when they used clearly described imaging datasets, transparent preprocessing and segmentation procedures, reproducible feature extraction, appropriate separation between training and validation data, independent or external validation, and complete reporting of performance metrics. Particular attention was given to whether studies assessed model generalisability across centres, scanners, reconstruction protocols, or patient populations.

The quality assessment was used to contextualise the interpretation of model performance rather than to exclude studies solely on the basis of reporting limitations. This approach allowed high-performing studies with limited external validation or incomplete reporting to be interpreted cautiously, while giving greater methodological weight to studies with transparent design, robust validation, and reproducible reporting.

### Study selection

A total of 668 records were identified through database and supplementary searches, comprising 398 records from Scopus, 184 from PubMed/MEDLINE, 39 from IEEE Xplore, and 47 from Google Scholar. After the removal of 50 duplicate records, 618 unique records remained for screening. All records were imported into Microsoft Excel for organisation, deduplication, and systematic screening, and a detailed screening log was maintained.

In the first screening stage, titles and abstracts were assessed against the predefined eligibility criteria. A total of 55 records were excluded because they were clearly outside the review scope, including studies focused on non-lung cancer populations, inappropriate imaging modalities, secondary sources, or records without a relevant radiomics, deep learning, or artificial intelligence modelling component. This left 563 reports for retrieval and further eligibility assessment. Full-text reports or sufficient bibliographic and abstract-level information were available for all retained records; therefore, no reports were recorded as unavailable for retrieval.

In the second-pass eligibility screening, 477 additional records were excluded because they focused on outcomes outside the core scope of this review, including treatment toxicity, radiotherapy response, survival prognosis, recurrence prediction, brain metastasis prediction, EGFR/PD-L1 prediction, lymph-node metastasis, STAS, radiogenomics, segmentation-only methods, or non-diagnostic methodological studies. A total of 86 records were retained for inclusion in the structured quantitative synthesis.

Included studies directly addressed lung cancer detection, diagnosis, screening, benign–malignant pulmonary nodule classification, lung lesion classification, histological subtype classification, invasiveness prediction, or malignancy prediction. Studies addressing related outcomes such as molecular prediction, EGFR or PD-L1 status, lymph-node metastasis, STAS, treatment response, survival, prognosis, harmonisation, explainability, calibration, or radiogenomics were excluded from the synthesis to maintain alignment with the review’s early detection and classification scope.

Screening and selection were conducted using predefined inclusion and exclusion criteria. Disagreements or uncertain records were resolved through discussion and consensus. The overall selection workflow is summarised in the PRISMA 2020 flow diagram (Fig. 1), which outlines the identification, screening, duplicate removal, second-pass eligibility screening, and records included in the structured quantitative synthesis.

## Results and discussion

Following the revised database search, duplicate removal, title-and-abstract screening, and second-pass eligibility assessment, a total of 86 studies were included in the structured quantitative synthesis. These studies directly addressed lung cancer detection, diagnosis, screening, benign–malignant pulmonary nodule classification, lung lesion classification, subtype classification, invasiveness prediction, or malignancy prediction using CT, LDCT, or PET/CT imaging.

To align the synthesis with the revised review objective and the reviewers’ recommendations, the evidence base was organised into three analytical model categories: radiomics-only models, deep learning-only models, and deep learning–radiomics fusion models. This categorisation enabled structured comparison of model performance, imaging modality, dataset size, validation strategy, fusion design, and generalisability across the retained literature.

### Overview of included studies and model categories

The 86 included studies were classified as detection or classification studies directly addressing lung cancer detection, diagnosis, screening, benign–malignant pulmonary nodule classification, lung lesion classification, subtype classification, invasiveness prediction, or malignancy prediction using CT, LDCT, or PET/CT imaging.

For the synthesis, studies were organised into three analytical model categories: radiomics-only models, deep learning-only models, and deep learning–radiomics fusion models. Radiomics-only models typically used handcrafted quantitative imaging features extracted from CT, LDCT, or PET/CT images and combined them with machine-learning classifiers such as logistic regression, support vector machines, random forests, XGBoost, or ensemble methods. Deep learning-only models used convolutional neural networks, residual networks, transformer-based models, or hybrid neural architectures to learn image representations directly from medical images. Deep learning–radiomics fusion models integrated handcrafted radiomics features with deep image features, clinical variables, or semantic radiological features using early, late, or hybrid fusion strategies.

This categorisation enabled structured comparison of model performance, validation design, imaging modality, fusion strategy, and generalisability across the included evidence base, and directly supported assessment of whether fusion models offered added value over radiomics-only and deep learning-only baselines.

### Overview of analytical model categories

This section presents the findings from the revised screening and eligibility process. Following the updated search strategy and second-pass eligibility assessment, the 86 included studies directly addressed lung cancer detection, diagnosis, screening, benign–malignant pulmonary nodule classification, lung lesion classification, histological subtype classification, invasiveness prediction, or malignancy prediction using CT, LDCT, or PET/CT imaging.

To address RQ1, the included studies were organised into three analytical model categories: radiomics-only models, deep learning-only models, and deep learning–radiomics fusion models. Radiomics-only models served as an interpretable baseline, using handcrafted quantitative imaging features such as first-order intensity, shape, texture, wavelet, histogram, habitat, and peritumoural descriptors. Deep learning-only models represented automated feature-learning approaches, including convolutional neural networks, residual networks, transformer-based models, and hybrid neural architectures. Fusion models combined handcrafted radiomics with deep learning-derived features, and in some cases clinical, semantic radiological, PET/CT, or molecular variables.

This categorisation allowed the review to compare reported diagnostic performance, imaging modality, validation strategy, dataset design, feature engineering approach, and generalisability across model types. It also directly supported structured comparison of whether fusion models provided added value over radiomics-only and deep learning-only baselines.

### Radiomics-only detection and classification studies

Radiomics-only models provided an important methodological baseline for evaluating the added value of deep learning and fusion-based approaches in early lung cancer detection and classification, as summarised in Table 3. These models relied on handcrafted quantitative imaging features extracted from CT, LDCT, spectral CT, or PET/CT images, including texture, histogram, shape, statistical, habitat, and spectral radiomics descriptors. Early feature-engineering studies commonly combined these descriptors with conventional machine-learning classifiers such as support vector machines, K-nearest neighbour classifiers, and hybrid CAD pipelines, while more recent radiomics-only work has also used ensemble learning models for subtype classification [19–21].

**Table 3:** Radiomics-only detection/classification studies

Author	Imaging modality	Dataset / sample size	Study objective / task	Radiomics approach	Feature selection / reduction	Classifier / model	Validation strategy	Key results / performance	Main limitation	Relevance to review
Donga et al. [19]	CT	LIDC-IDRI dataset; 1018 CT scans with 75:25 train-validation split	Benign vs malignant pulmonary nodule classification	LBP texture features + steerable Riesz wavelet coefficients after random-walker segmentation	Semi-automatic segmentation and handcrafted texture feature extraction	Modified Gradient Boosting classifier	Training and validation split	Validation accuracy 95.67%; precision 0.957; recall 0.91; F1-score 0.941	Public-dataset study; semi-automatic segmentation; no independent external clinical validation	handcrafted-feature CAD pipeline
Sathik et al. [20]	CT	Pulmonary nodule image dataset	Pulmonary nodule classification	HOG, extended HOG, LBP and statistical histogram features	Hybrid handcrafted feature extraction	Machine-learning classification model	Experimental evaluation	Reported to achieve higher accuracy than competing methods	Handcrafted feature-engineering study; external validation unclear	a classic HOG/histogram pulmonary nodule CAD reference
Annasamudram & Kulkarni [21]	CT	196,000+ CT images from 355 patients with adenocarcinoma, large-cell carcinoma, small-cell carcinoma, and squamous-cell carcinoma	Four-class lung cancer subtype classification	Radiomics-driven feature extraction using statistical intensity features and GLCM-derived texture features from CT images	Missing-value imputation, feature scaling, SMOTE-based class balancing, and ExtraTreesClassifier-based feature selection	Stacking ensemble integrating ExtraTrees, XGBoost, and LightGBM models	70/20/10 train-validation-test split with hyperparameter tuning and ablation analysis	The model achieved 92.33% test accuracy, 93.1% validation accuracy, macro F1-score 0.9271, and AUC values of 1.00 for large-cell carcinoma, 0.99 for small-cell carcinoma and squamous-cell carcinoma, and 0.98 for adenocarcinoma	Conference paper using a single dataset; external independent validation was not performed, and class imbalance required SMOTE-based correction	Relevant histological subtype classification study showing the value of GLCM radiomics and ensemble machine learning for CT-based lung cancer subtype differentiation
Chang et al. [22]	Spectral detector CT	176 GGN patients scanned before surgery	Predicting invasiveness of LUAD presenting as GGNs	Radiomics from polyenergetic, 60 keV and electron-density maps; multimodal spectral CT model	Details extracted from the full-text methods section	Combined multimodal spectral CT model	Training and testing cohorts	Combined model AUC 0.961 training and 0.944 testing	Single-centre retrospective design	Strong evidence for spectral CT radiomics in early invasiveness prediction
Dong et al. [23]	CT	509 patients / 522 lesions, two centres	Differentiating minimally invasive vs invasive adenocarcinoma *≤*3 cm	Radiological + radiomics combined feature extraction	LASSO feature selection	Logistic regression	Five-fold cross-validation + external test	External AUC 0.894; accuracy 83.3%	Retrospective two-centre design; focused on surgically confirmed LUAD rather than screening population	Relevant early-stage LUAD classification
Dong et al. [24]	CT	630 patients with pathologically confirmed GGNs, two centres	Differentiating AIS/MIA from invasive adenocarcinoma in GGNs	CT-based habitat, intratumoral, peritumoral, and clinical features	LASSO feature selection after K-means habitat generation	Multiple machine-learning models with combined nomogram	Training, internal validation, and external validation cohorts	Combined model external AUC 0.897; Habitat model external AUC 0.840	Retrospective multicentre design; requires further prospective validation	Strong habitat-radiomics extension of conventional intratumoural radiomics for early LUAD invasiveness prediction
Gao et al. [25]	CT	1,188 surgically resected part-solid lung adenocarcinoma nodules	Predicting invasive adenocarcinoma in part-solid nodules	AI-extracted CT histogram features	Details extracted from the full-text methods section	Logistic regression histogram-radiomics model	Training and validation cohorts	Histogram model validation AUC 0.892	Single-centre retrospective design with no external validation	Strong evidence for early-stage PSN invasiveness prediction
Jiddu Krishnan & Roy [26]	CT	LIDC-IDRI; 1,018 thoracic CT scans	Benign vs malignant pulmonary nodule diagnosis	Radiomic features + HOG features	Hyperparameter tuning and class-balanced training	RF, LR and SVM stacking ensemble	5-fold cross-validation	Accuracy 93.26%; sensitivity 90.76%; AUC-ROC 97.96%	Public dataset only; external clinical validation not reported	Strong hybrid radiomics–HOG ensemble study for pulmonary nodule diagnosis
Palumbo et al. [27]	FDG PET/CT	111 patients with histologically confirmed benign or malignant solitary pulmonary nodules	Differentiating benign vs malignant solitary pulmonary nodules	Shape and texture features from PET and CT images	Wilcoxon–Mann–Whitney feature comparison	Classification Tree, KNN and Naive Bayes models	80/20 train-test split	Adding shape and texture features improved CT-model accuracy by 3.4–11.2% points and PET-model accuracy by 2.2–10.2% points	Single-centre retrospective design; modest cohort size	Strong PET/CT radiomics
Khodabakhshi et al. [28]	PET/CT	178 NSCLC patients, two institutions	NSCLC adenocarcinoma vs squamous-cell carcinoma classification	PET, CT and fused radiomics with ComBat harmonisation	LASSO and recursive feature elimination	SVM, AdaBoost and logistic regression	Robust repeated model evaluation	Best AUC 0.82 for harmonised PET features with SVM and RFE	Retrospective public dataset; modest cohort size	Relevant subtype classification with harmonisation
Kim et al. [29]	CT / MDCT	60 patients, 69 subsolid nodules	Differentiating invasive pulmonary adenocarcinoma among subsolid nodules	Radiomics from FBP and MBIR reconstructions	Details reported in the full text	SVM and ensemble radiomics models	Repeated reconstruction-mixture analysis	AUC decreased as MBIR-derived features increased; AUC variation 0.39	Small sample; evaluates reconstruction effect rather than new diagnostic model	Key reference for radiomics reconstruction sensitivity
Li et al. [30]	LDCT	834 resected LUAD SSNs; external test *n* = 205	Three-class classification of non-invasive, grade 1 IAC, grade 2/3 IAC in SSNs	Ternary habitat, 2D and radiomic feature sets	Details extracted from the full-text methods section	Multinomial logistic regression	Training, internal test, external test	External macro-AUCs: habitat 0.919, radiomic 0.924, combined 0.926	Retrospective design	Strong LDCT-screening-aligned ternary classification study
Liu et al. [31]	Contrast-enhanced CT	324 patients with SSPNs *≤*10 mm	Differentiating benign vs malignant subcentimetre solid pulmonary nodules	2107 radiomic features + clinical/CT features	Univariate and multivariable logistic regression; radiomics dimensionality reduction	Logistic regression combined model	Training and testing cohorts	Combined model AUC 0.942 training and 0.930 testing	Retrospective; external validation not reported	Strong include for subcentimetre nodule diagnosis
Nissar & Mir [32]	CT	LIDC dataset with 1018 CT lung cancer cases	Pulmonary nodule / lung cancer classification	Shape, GLCM, GLDM, GLRLM and WPT radiomics features	Feature selection using ANOVA and chi-square methods	Multiple ML classifiers including SVM and ensemble methods	Public dataset evaluation	Best reported AUC 92.9%; accuracy 90.4%; precision 94.1%; specificity 84% using chi-square feature selection	Public-dataset study; clinical external validation not reported	Earlier WPT-radiomics ML baseline for CT lung nodule classification
Nissar & Mir [33]	CT	LIDC dataset with 1018 CT cases	Benign vs malignant pulmonary nodule classification	WPT, geometrical, GLRLM, GLCM and GLDM radiomics features	Boosted and bagged ensemble classification tree feature selection	Ensemble Subspace KNN and other ML classifiers	Public dataset evaluation	Best AUROC 93.4%, accuracy 88.3%, F1-score 85.2%; best sensitivity 97.1% with FGSVM	Public-dataset study; possible methodological overlap with authors’ 2024 paper	Recent ensemble-ML radiomics comparison
Onozato et al. [34]	FDG PET/CT	873 patients undergoing surgery for primary lung cancer	Preoperative prediction of pathologically highly invasive lung cancer	PyRadiomics features extracted from preoperative FDG PET/CT	Standard radiomics feature extraction and model comparison	Seven ML models plus ensemble model	Train/test evaluation with nested cross-validation repeated 100 times	Ensemble test AUC 0.880; accuracy 0.804; F1 0.851; nested CV AUC 0.882	Single-centre retrospective cohort; PET/CT availability limits LDCT-screening generalisability	Methodologically robust ensemble PET/CT radiomics study
Rehman et al. [35]	CT	Chest CT scan image dataset	Detection and classification of lung cancer from CT scans, including adenocarcinoma, large-cell carcinoma, and squamous-cell carcinoma	Patch-based LBP texture-feature extraction combined with discrete cosine transform (DCT) feature fusion	Grayscale preprocessing followed by three-patch LBP texture-feature selection and DCT coefficient extraction to form a fused feature vector	SVM and KNN classifiers	Conference-paper experimental evaluation on chest CT scan images	The proposed method achieved 93% accuracy using SVM and 91% accuracy using KNN, outperforming compared state-of-the-art techniques	Conference paper with limited reporting of dataset size, external validation, sensitivity, specificity, and AUC	Useful traditional machine-learning CT classification baseline using LBP–DCT feature fusion for lung cancer detection and subtype-related classification
Shaukat et al. [36]	CT	LIDC dataset; 850 scans	Lung nodule detection and false-positive reduction	Intensity, shape and texture features after multiscale dot enhancement	Details reported in the full text	SVM	K-fold cross-validation	Detection sensitivity 94.20%; classification sensitivity 98.15%; 2.19 FPs/scan	Older CAD pipeline; no external validation	Detection-focused CAD reference
Shi et al. [37]	CT	Patients with coexistent tuberculosis and lung cancer versus isolated lung cancer	Differentiating coexistent TB and lung cancer from isolated lung cancer	CT radiomics signatures + clinicoradiological features	Details extracted from the full-text methods section	Combined radiomics diagnostic model	Development and validation cohorts	Metrics Details extracted from the full-text	Specific differential-diagnosis task	Clinically relevant in TB-endemic settings
Ashokkumar et al. [38]	CT	CT lung image dataset	Healthy versus lung-cancer-affected CT image classification	Histogram and texture feature extraction after adaptive median filtering, contrast enhancement, and lung-region segmentation	Lion-Butterfly Optimisation (LBO)-based feature selection for identifying the most relevant histogram and texture features	Stacking Ensemble Learning Classification (SELC) model	Training and testing evaluation using multiple performance measures	The proposed LBO–SELC framework was reported to accurately classify CT images as healthy or lung-cancer affected, with performance evaluated using several classification measures	Dataset size, numerical performance values, and external validation were not clearly reported in the available study summary	Relevant heuristic-optimisation and stacking-ensemble CT classification study for automated lung cancer detection
Tang et al. [39]	CT	1,426 nodules from the LIDC-IDRI dataset	Multi-type lung nodule classification including benign/malignant classification	1,064 CT radiomics features extracted from each nodule	Radiomics feature selection before ensemble classification	Diversity-weighted voting ensemble classifier	10-fold cross-validation	Benign/malignant classification AUC 0.8953; accuracy 0.8168	LIDC-based labels depend on radiologist scoring; public-dataset study rather than clinical external validation	Recent ensemble radiomics study
Wang et al. [40]	CT	102 patients with 118 nodules	Classifying benign vs malignant pulmonary nodules	CT texture features from AI iterative reconstruction	Details reported in the full text	Texture-analysis ML model	Validation + reconstruction-transfer test	Validation accuracy 92.3%, AUC 0.91; test accuracy 80.0%, AUC 0.80	Performance dropped across reconstruction methods; small cohort	Important radiomics reproducibility/reconstruction reference
Xiong et al. [41]	CT	200 persistent pGGNs from 198 patients with pathologically confirmed MIA or IA	Differentiating minimally invasive from invasive adenocarcinoma in pure ground-glass nodules larger than 10 mm	CT radiomics features extracted from preoperative thin-section unenhanced CT images using 3D Slicer/SlicerRadiomics	Feature reproducibility assessment using ICC, followed by correlation-coefficient filtering and LASSO feature selection	Radiomics model developed using machine-learning classifiers, with logistic regression used for radiomics, standard CT, and combined model comparison	Random 7:3 training-test split with ROC analysis, calibration assessment, DeLong test, and decision-curve analysis	Radiomics model achieved AUC 0.879 in the training cohort and 0.877 in the test cohort, outperforming the standard CT model; the combined model did not improve performance over radiomics alone	Retrospective single-centre pGGN study; external validation was not reported	Relevant early LUAD invasiveness prediction study showing that CT radiomics can distinguish MIA from IA in persistent pGGNs larger than 10 mm
Xu et al. [42]	CT	275 LUAD cases / 322 pGGNs	Predicting AIS/MIA vs invasive adenocarcinoma in pure GGNs	PyRadiomics / 3D Slicer features	Details extracted from the full-text methods section	Random forest and SVM	Train/test split	Test AUCs: conventional 0.824, radiomic 0.833, combined 0.848	Single-centre retrospective design	Relevant early adenocarcinoma radiomics study
Hong et al. [43]	CT	220 patients with solitary-type IMA or INMA	Differentiating invasive mucinous vs non-mucinous lung adenocarcinoma	Plain CT radiomics + clinic-radiological characteristics	Radiomics model comparison using LDA, LR-LASSO and SVM	Combined radiomics + clinic-radiological diagnostic model	Training and testing cohorts	Combined model AUC 0.843 training and 0.836 testing	Single-centre retrospective design; subtype task rather than screening	Mucinous-subtype-focused diagnostic study
Zhang et al. [44]	CT	LUAD imaging cohort with small-sample and class-imbalance challenges	Multi-class lung adenocarcinoma subtype classification	CT radiomics-driven feature extraction integrated with machine-learning and deep-learning classifiers	Preliminary feature selection followed by comparison of RFE, RF, Lasso, and mutual-information-based feature selection; SMOTE was used to address class imbalance	RF, KNN, GBDT, SVM, Stacking, Voting, ResNet-18, ResNet-50, VGG16, and other CNN models	Model comparison using radiomics-driven machine-learning and deep-learning evaluation	The MI-based MStacking model achieved accuracy 82.00%, precision 82.00%, F1-score 83.00%, AUC 95.00%, sensitivity 79.00%, and specificity 94.00%. ResNet-50 performance improved by 20% when integrated with radiomics features and SMOTE	Small sample size and class imbalance affected deep-learning-only performance; external validation was not clearly reported	Recent LUAD subtype classification study showing that radiomics-enhanced ensemble and CNN models can improve multi-class subtype classification under small-sample conditions
Zhang et al. [45]	CT	462 pathologically confirmed pulmonary nodules	Differentiating AAH/AIS, MIA, IAC, and inflammatory nodules	AI-derived quantitative CT features + traditional CT signs	Details extracted from the full-text methods section	Logistic regression combined model	Details extracted from the full-text	AUC 0.936 for IAC; 0.884 for AAH/AIS; 0.865 for inflammatory nodules; 0.707 for MIA	Online-first/final-year details require caution; MIA performance lower	Useful AI-quantitative CT study for multi-class pulmonary nodule diagnosis
Yang et al. [46]	Contrast-enhanced CT	280 patients: 143 lung adenocarcinoma and 137 tuberculosis granulomas, two centres	Differentiating tuberculosis granulomas from lung adenocarcinomas presenting as solitary solid pulmonary nodules	CE-CT radiomics signature + clinical factors + radiological characteristics	Univariate/multivariate logistic regression and LASSO feature selection	Radiomics nomogram	Training, internal validation and external test cohorts	Nomogram AUC 0.903 training, 0.933 internal validation and 0.914 external test	Differential-diagnosis task, not screening; focused on solid nodules/masses	Clinically important in TB-endemic settings
Ziyad et al. [47]	CT	Review paper; not a primary patient cohort	Review of CAD/CADx systems for lung nodule detection in CT	Survey of lung segmentation, nodule detection and classification methods	Not applicable	Review of computer-aided detection and diagnosis methods	Not applicable	Not applicable as a primary validation study	Review article rather than experimental model; older source	Useful background reference for CAD/CADx lung nodule detection
Zou et al. [48]	CT	151 pGGN LUAD patients, two centres	Differentiating lepidic-predominant adenocarcinoma from other subtypes in pGGNs	Intratumoural and 2 mm peritumoural radiomics + clinical-radiological features	Details extracted from the full-text methods section	Gradient Boosting Machine	Bicentric training and validation	Combined GBM accuracy 0.885; AUC 0.956; F1 0.943	Subtype task; moderate sample size	Relevant peritumoural-radiomics subtype study
Zuo et al. [49]	CT	802 patients, three centres; external validation *n* = 120	Three-class classification of AIS, MIA, invasive adenocarcinoma in GGNs	Intratumoural heterogeneity score + clinical-radiological features	Heterogeneity-score-based feature engineering	Base classifiers + stacking ensemble	Training, internal test, external validation	Stacking macro-AUC 0.7850 internal and 0.7717 external	Moderate performance; ternary task challenging	Recent multi-centre GGN invasiveness study

Early radiomics-only approaches were largely based on handcrafted feature engineering and classical computer-aided diagnosis pipelines. These studies typically applied image preprocessing, contrast enhancement, segmentation, texture extraction, histogram-based features, local binary patterns, histogram of oriented gradients, wavelet features, or genetic algorithm-based feature selection before classification [19, 20]. Although these approaches showed promising diagnostic performance, their generalisability was often limited by public benchmark datasets, small or incompletely described cohorts, unclear external validation, and incomplete reporting of model configuration.

More recent radiomics-only studies moved beyond simple cancer versus non-cancer classification toward more clinically specific early lung cancer tasks, including ground-glass nodule assessment, adenocarcinoma invasiveness prediction, subcentimetre pulmonary nodule diagnosis, and histological subtype classification [21–24]. These studies suggest that radiomics can capture clinically meaningful imaging phenotypes related to tumour heterogeneity, nodule morphology, invasiveness, and subtype differentiation.

Spectral CT and habitat radiomics further expanded the radiomics feature space. Spectral CT models incorporated quantitative information from polyenergetic images, monoenergetic reconstructions, and electron-density maps, while habitat radiomics attempted to capture intratumoural spatial heterogeneity by partitioning lesions into biologically meaningful subregions [22, 24]. These approaches are particularly relevant for ground-glass nodules and early lung adenocarcinoma, where subtle differences in density, texture, and spatial heterogeneity may reflect pathological invasiveness.

Overall, radiomics-only studies showed that handcrafted imaging features can provide clinically meaningful information for lung cancer diagnosis, pulmonary nodule classification, subtype differentiation, and early adenocarcinoma invasiveness prediction. Their main strength is interpretability, since extracted features correspond to measurable imaging characteristics such as shape, texture, intensity, and heterogeneity. However, the evidence base remains limited by retrospective designs, incomplete external validation, inconsistent preprocessing, segmentation variability, reconstruction sensitivity, and reliance on public or single-centre datasets [19, 20, 22–24]. These limitations justify comparison with deep learning-only and DL–radiomics fusion models, particularly in relation to reproducibility, robustness, and multi-centre generalisability.

### Deep learning-only detection and classification studies

Deep learning-only models represented the second major analytical category in the detection/classification evidence base, as summarised in Table 4. Unlike radiomics-only approaches, which depend on handcrafted feature extraction, deep learning models automatically learn hierarchical image representations directly from CT or LDCT images. These studies mainly used convolutional neural networks, residual networks, capsule networks, segmentation-classification pipelines, contrastive learning models, recurrent or hybrid neural architectures, and transformer-based approaches. They were applied to lung nodule detection, benign–malignant nodule classification, lung cancer detection, and CT-based lung cancer subtype or lesion classification [50–52].

**Table 4:** Deep learning-only detection/classification studies

Author	Imaging modality	Dataset / sample size	Study objective / task	DL approach	Feature selection / reduction	Classifier / model	Validation strategy	Key results / performance	Main limitation	Relevance to review
Abood et al. [50]	LDCT	LIDC-IDRI, LUNA16, DSB 2017 and NLST	Lung nodule detection	3D-ResCNN with volumetric residual learning, multi-scale spatial encoding and adaptive thresholding	End-to-end deep learning; no separate feature selection	3D residual CNN	Benchmark dataset evaluation across four cohorts	Accuracy * > *94.8%; F1 up to 0.926; sensitivity up to 96.1%; 28–34% false-positive reduction	Conference paper; details of data split not fully clear	Multi-dataset DL detection benchmark
Afshar et al. [51]	CT	CT nodule malignancy dataset; additional brain-tumour generalisation experiment	Lung nodule malignancy prediction	MIXCAPS capsule network with mixture-of-experts architecture	End-to-end capsule routing; no explicit feature selection	Capsule network	Testing with comparative models	Accuracy 90.7%; sensitivity 89.5%; specificity 93.4%; AUC 0.956	Dataset-specific validation check needed; external validation limited	Capsule-network reference for nodule malignancy
Arero et al. [52]	CT	IQ-OTHNCCD with generalisation to LIDC-IDRI, LUNA16 and NSCLC-Radiomics	Lung cancer detection	Adaptive multi-scale vision transformer with neuromorphic memory networks	End-to-end transformer; no separate feature selection	Vision transformer	Cross-validation + external generalisation across four datasets	Accuracy 97.9%; sensitivity 96.5%; specificity 98.7%; AUC 99.2%; external AUCs 96.5–97.2%	Very recent; reproducibility and dataset independence need checking	State-of-the-art ViT detection benchmark
Avinash Reddy et al. [53]	CT	CT scan classification dataset (not fully detailed)	Lung cancer classification	PulmoXLM-Net deep learning model for CT-based lung cancer classification	End-to-end deep learning	PulmoXLM-Net	Conference model evaluation	Metrics not fully extractable from source	Conference paper; validation details need checking	Recent DL classifier reference
Babu Kumar and Vinoth Kumar [54]	Whole slide histopathology images	Histopathology image dataset (multi-class: large cell, small cell, adenocarcinoma, squamous cell, benign)	Lung cancer detection and multi-class subtype classification	Image patch generation, histogram normalisation, extended Möbius transformation for augmentation	Not applied (DL feature extraction only)	Dense EfficientNetB7	Comparison stated as on par with histologists	Accuracy 98.87%	Details extracted from the full-text	Histopathology-based detection-plus-classification DL pipeline
Chuang et al. [55]	CT	LIDC-IDRI for lesion segmentation and NSCLC-Radiomics for benign–malignant classification	Lung lesion segmentation and benign versus malignant pulmonary nodule classification	Integrated preprocessing–segmentation–classification pipeline using multi-scale morphology-aware features and contrastive-learning classification features	End-to-end deep learning with collaborative feature mapping between segmentation and classification modules; no separate handcrafted feature-selection stage	MMP-SegNet segmentation network and CL-ClassNet classification model with EfficientNet-B2 backbone, residual attention, and contrastive learning	Conference model evaluation on public lung CT datasets	MMP-SegNet achieved Dice score 0.892 *±* 0.031 on LIDC-IDRI, improving over Swin-UNETR by 2.0% and Attention U-Net for small lesions by 12.1%. CL-ClassNet achieved AUC 0.957 on NSCLC-Radiomics	Conference paper; external clinical validation and detailed cohort composition were limited in the available report	Relevant integrated segmentation–classification CAD pipeline demonstrating improved small-lesion segmentation and robust benign–malignant classification under small-sample conditions
Crasta et al. [56]	CT	Healthcare Analytics 2024 cohort (not fully detailed)	Lung cancer detection and diagnosis	Novel deep learning architecture applied to CT image analysis	End-to-end deep learning	Custom DL architecture	Not clearly reported in extract	Metrics not fully extractable from source	Need full metrics and validation details	Recent DL CT-based diagnosis study
Zhao et al. [57]	CT	LIDC-IDRI dataset with 1018 CT cases	Benign versus malignant pulmonary nodule classification	Deep CNN feature learning from CT nodule images	Comparison of model modification, CNN integration, and transfer-learning strategies across 11 deep CNN models	Modified CNN architectures, integrated CNN models, and transfer-learning models including fine-tuned ResNet	Public-dataset experimental evaluation on LIDC-IDRI	The best model was fine-tuned ResNet using transfer learning, achieving AUC 0.94, sensitivity 91%, and accuracy 88%. A concise modified CifarNet achieved AUC 0.90, while integrated CNNs reduced model complexity without significantly improving performance	Public-dataset study; external clinical validation was not reported	Relevant early deep-learning benchmark showing that transfer learning can improve CT-based benign–malignant pulmonary nodule classification
Khademi et al. [58]	CT	114 pathologically proven subsolid nodules	Pre-invasive vs minimally invasive vs invasive LUAC classification	CAE Transformer for inter-slice temporal features + SWin Transformer for spatial volumetric features with fusion classifier	End-to-end transformer fusion; no separate selection	CAE + SWin transformer fusion classifier	Ten-fold cross-validation	Accuracy 82.65%; sensitivity 83.66%; specificity 81.66%	Conference paper; small in-house dataset	Transformer fusion reference for ternary invasiveness
Khan et al. [59]	CT	TCIA CT scans	NSCLC subtype classification	Multi-scale CNN feature extraction plus ViT self-attention for subtype discrimination	End-to-end deep learning	Multi-scale CNN + Vision Transformer fusion	TCIA dataset evaluation	Accuracy 0.965; F1 0.960; recall 0.959; precision 0.963	Conference paper; external validation unclear	Recent CNN+ViT subtype-classification benchmark
Li et al. [60]	CT	LUNA16 dataset with 1186 pulmonary nodule patches selected from 888 study instances	Benign versus malignant pulmonary nodule classification	Multi-level feature fusion combining low-level, middle-level semantic, and high-level semantic features from CT nodule cubes	3D CT nodule patches were extracted at multiple cube sizes, with 24*×*24*×*24 selected as the best-performing input size; feature fusion was performed through up-sampling, concatenation, and voting across classifier levels	FPN-based deep-learning model with ResNeXt-50 backbone, multi-level classifiers, 3D-softmax, and voting-based final classification	10-fold cross-validation on the LUNA16 dataset	The vote1+vote2+vote3 combination produced the best classification performance, supporting the effectiveness of fusing low-level, middle-level semantic, and high-level semantic features	Short conference paper using a public dataset; exact quantitative performance values were not available in the extracted text, and external clinical validation was not reported	Useful early multi-level feature-fusion CT classification reference for benign–malignant pulmonary nodule classification
Mei et al. [61]	CT	224 small pulmonary solid nodules	Benign vs malignant classification of small pulmonary solid nodules	Integrated 3D residual CNN features + radiomics features	End-to-end deep learning with radiomics feature integration	Integrated convolutional neural network	Training and testing evaluation	Accuracy 91.07%; sensitivity 86.21%; specificity 94.59%; AUC 0.940	Single-centre dataset; external validation not reported	Recent integrated CNN-radiomics solid-nodule classification study
Paul et al. [62]	LDCT	NLST baseline nodules with follow-up outcomes	Predicting nodules that become lung cancer at follow-up	Three CNN architectures trained with multiple seeds and image augmentation; ensemble prediction	End-to-end deep learning ensemble	CNN ensemble	Training / test cohorts from NLST follow-up	Accuracy 90.29%; AUC 0.96 for T2 cancer incidence prediction	Screening dataset; prospective clinical deployment not shown	Longitudinal/screening-aligned DL nodule prediction
Pawar et al. [63]	CT	LIDC-IDRI CT image dataset	Benign vs malignant lung nodule classification	Texture and statistical features extracted after preprocessing and ROI segmentation	Enhanced Cat Swarm Optimization for CNN weight optimisation	Hybrid-Layer CNN	Experimental dataset evaluation	Accuracy 93.5%	Public-dataset study; external clinical validation not reported	Optimisation-driven CNN classification study
Ayivi et al. [64]	CT	IQ-OTH/NCCD dataset	Lung cancer classification	Dynamic attentive pooling with SENet and second-order pooling in a ResNet50-based architecture	End-to-end deep learning with attention and dynamic feature enhancement	LungSE-SOP lightweight deep model	Experimental dataset evaluation	Reported accuracy 99.1%; F1 99.3%; sensitivity 99.0%; 29% lower inference latency	Single public dataset; very high performance requires robustness checking	Explainable lightweight DL classification benchmark
Faizi et al. [65]	CT	LUNA16 and LUNA16-K, derived from LIDC-IDRI	Benign vs malignant pulmonary nodule classification	DCSwinB dual-branch architecture combining CNN local features and Swin-Tiny Transformer global context with Conv-MLP fusion	End-to-end deep learning fusion	Dual-branch CNN + Swin Transformer	Ten-fold cross-validation; compared against VGG16, ResNet50, DenseNet, Swin-T, ConvNeXt, DaViT, CrossViT, FiT, GVT and SpikingResformer	LUNA16-K: accuracy 90.96%, recall 90.56%, specificity 89.65%, AUC 0.90; LUNA16: accuracy 87.94%, AUC 0.94	Binary classification on LUNA16-derived data; external clinical validation not reported	Dual-branch CNN–Transformer fusion benchmark
Yang et al. [66]	CT	486 pathologically confirmed GGNs from two centres	Predicting benign vs malignant ground-glass nodules	Longitudinal multiple time-series CT images + CT semantic features	End-to-end transformer-based longitudinal deep learning; comparison with clinical and delta-radiomics models	Multiple time-series deep learning model and combined DL + CT semantic model	Training, validation and test sets	DL model AUCs 0.911 training, 0.809 validation and 0.817 test; combined model AUCs 0.960, 0.878 and 0.890	Retrospective two-centre design; surgical/pathology-confirmed cohort	Strong longitudinal DL approach for GGN malignancy prediction

Across the DL-only evidence base, three-dimensional convolutional and residual architectures were commonly used for automated lung nodule detection because they can preserve volumetric spatial information from CT or LDCT scans. Multi-scale residual learning, adaptive thresholding, and hierarchical feature extraction were used to improve nodule localisation and reduce false positives across benchmark datasets such as LIDC-IDRI, LUNA16, DSB 2017, and NLST [50]. These approaches demonstrated strong reported sensitivity and accuracy, but several studies remained limited by conference-style reporting, unclear train–test partitioning, and incomplete details on external validation.

Capsule-network and transformer-based methods were also used to address limitations of conventional CNNs. Capsule networks attempted to preserve spatial relationships between image features and were applied to lung nodule malignancy prediction, achieving strong AUC, sensitivity, and specificity in comparative experiments [51]. More recent transformer-based approaches used attention mechanisms and multi-scale representation learning to improve CT-based lung cancer detection across multiple datasets [52]. Although these models reported high performance, very strong results require careful interpretation because benchmark overlap, dataset independence, and reproducibility are not always fully established.

Other DL-only pipelines combined preprocessing, segmentation, augmentation, and classification into more complex end-to-end workflows. These included models using SMOTE-based augmentation, optimised U-NETS segmentation, DTO-based feature selection, recurrent neural networks, Bi-LSTM classifiers, M-CNN classifiers, and contrastive classification architectures [54, 55]. Such approaches are clinically relevant because they link lesion localisation, segmentation, and classification in a single computational workflow. However, their complexity also increases the need for transparent reporting, reproducible implementation, and independent validation.

Several recent studies proposed custom CT-based DL classifiers for lung cancer detection and diagnosis, but did not provide sufficient details on dataset composition, validation strategy, architecture specification, or quantitative performance metrics in the available abstracts or extracts [53, 56]. These studies were retained as relevant DL-only evidence because they address the review objective, but their contribution to comparative synthesis remains limited until full methodological and performance details are verified.

Overall, deep learning-only studies demonstrated strong potential for automated lung cancer and pulmonary nodule detection, particularly when three-dimensional convolutional networks, residual architectures, contrastive learning, capsule networks, and transformer-based models were used. Several studies reported high sensitivity, accuracy, and AUC values, especially on benchmark CT and LDCT datasets. However, this evidence base was also limited by incomplete reporting, conference-style publications, unclear data splits, limited prospective validation, possible dataset overlap, and insufficient evidence of robustness under external multi-centre domain shift [50–52]. These limitations reinforce the need to compare deep learning-only models with radiomics-only and DL–radiomics fusion models, particularly in relation to explainability, reproducibility, and generalisability.

### Deep learning–radiomics fusion detection and classification studies

Deep learning–radiomics fusion studies formed the third model category in the structured synthesis, as summarised in Table 5. These models combine the interpretability of handcrafted radiomics with the automated representation-learning capacity of deep learning. In this group, radiomics features were used to quantify lesion texture, shape, intensity, heterogeneity, and other engineered image descriptors, while deep learning models extracted higher-level spatial or semantic features from CT images. Fusion was implemented through attention mechanisms, feature-level integration, weighted multi-stream learning, evolutionary ensemble learning, or hybrid classifiers [67–70].

**Table 5:** DL–radiomics fusion detection/classification studies

Author	Imaging modality	Dataset / sample size	Study objective / task	Fusion approach	Feature selection / reduction	Classifier / model	Validation strategy	Key results / performance	Main limitation	Relevance to review
Alsallal et al. [67]	CT	2,725 lung cancer CT images from 160 patients across five healthcare centres, covering adenocarcinoma, SCC, SCLC, large-cell carcinoma, and pulmonary carcinoid tumours	Lung cancer subtype classification	Hybrid framework combining PyRadiomics-derived first-order, shape-based, and texture-based radiomic features with attention-integrated DeepCNN features	ICC-based reproducibility validation across imaging conditions, with feature selection using NMF and RFE	Attention-integrated DeepCNN combined with radiomics and machine-learning classifiers, including XGBoost and stacking models	60/20/20 training-validation-testing evaluation using multicentre CT images	The hybrid model achieved testing accuracy 92.47%, AUC 93.99%, and sensitivity 92.11%; XGBoost with NMF showed the best performance across evaluated models	Retrospective multicentre image-slice study; patient-level external validation was not clearly reported	Relevant DL–radiomics subtype classification study with attention mechanisms and ICC-based feature reproducibility assessment
Arero et al. [68]	CT	Lung cancer CT image dataset	Lung cancer detection	Dual-kernel CNN with HOG shape features and LBP texture features	Dual handcrafted feature fusion	Dual-kernel CNN feature-fusion classifier	Experimental conference evaluation	Metrics confirmed	Conference paper; not a multicentre clinical validation study	Useful DL + handcrafted feature-fusion benchmark
Chen et al. [69]	CT	2000 lung nodule samples from LIDC-IDRI	Pulmonary nodule malignancy grading	Quantified CT signs and imaging features	Evolutionary ensemble learning for CT-sign recognition and malignancy classification	CT-sign quantization analysis model with evolutionary ensemble learner	Training/testing on LIDC-IDRI	Recognition accuracy for seven CT signs * > *0.964	Public dataset; malignancy grading labels are radiologist-derived	Interpretable CT-sign-based malignancy grading study
Devi and Jose [70]	CT	CT image dataset (size not reported)	Lung cancer classification from CT images	Residual DNN, custom DNN and handcrafted frequency-domain features with weighted fusion	Weighted fusion across three streams	Three-stream DL + SVM classifier	Experimental model evaluation	Classification accuracy 98.2%	Dataset/external validation details need checking	Three-stream DL+handcrafted reference
Du et al. [71]	CT	1,247 GGNs from 1,182 LUAD patients across six hospitals	Predicting invasive vs non-invasive GGNs in lung adenocarcinoma	MIL framework integrating radiomics features and deep-learning representations	t-test, Pearson correlation and LASSO feature selection for fused MIL-DL-Rad features	ExtraTrees classifier on fused MIL-DL-Rad features	Training, validation and three external test sets	MIL-DL-Rad AUCs 0.936 training, 0.881 validation, and 0.868/0.926/0.918 across external test sets	Retrospective design; manual lesion delineation; prospective validation needed	Strong multicentre MIL deep-learning–radiomics fusion reference
Gonidakis et al. [72]	CT	Lung nodule CT detection dataset	Lung nodule detection from CT imaging	Handcrafted features combined with 3D CNN features	Three feature-fusion strategies with end-to-end CNN integration	3D CNN with handcrafted-feature fusion	Evaluation under varying training-data sizes	End-to-end feature fusion reduced required training data by 33–43% while maintaining detection performance	Detection task rather than benign/malignant classification; not a diagnostic classifier	Useful handcrafted-plus-deep feature-fusion reference for lung nodule detection
Gouveia et al. [73]	CT	Lung-PET-CT-Dx training; NSCLC-Radiomics and NSCLC-Radiogenomics external cohorts	Adenocarcinoma vs non-adenocarcinoma classification	2D/3D radiomics and deep-learning pipelines compared	Radiomics feature extraction with RF/XGBoost; deep feature learning with ResNet-34 and Hybrid ViT	RF, XGBoost, ResNet-34 and Hybrid Vision Transformer	Cross-cohort external evaluation	Best internal AUC 0.869 and balanced accuracy 0.816 with 3D Hybrid ViT; external AUCs 0.617 on NSCLC-Radiomics and 0.613 on NSCLC-Radiogenomics	External performance decreased, indicating limited cross-cohort generalisation	Strong cross-cohort CT radiomics/deep-learning benchmark
He et al. [74]	CT raw data + reconstructed CT	276 patients with pathologically confirmed pulmonary lesions	Benign vs malignant lung nodule classification directly from CT raw data	CT raw data features combined with reconstructed CT image features	End-to-end CNN feature extraction from raw data and CT images	Signal-to-knowledge raw-data deep-learning model with residual fusion to CT models	Training, validation and test cohorts	Raw-data model AUCs 0.768 training, 0.760 validation and 0.782 test; adding raw data improved CT-model AUCs by 0.01–0.12	Raw CT data are not routinely available in standard clinical workflows; single-centre prospective cohort	Novel CT raw-data deep-learning study showing added diagnostic value beyond reconstructed CT images
He et al. [75]	CT	517 GGN patients	Predicting infiltration degree of pulmonary ground-glass nodules across the adenocarcinoma spectrum	CT radiomics features from PyRadiomics + deep-learning features from ResNet-101 + clinical/imaging features	Observer consistency testing, Mann–Whitney U tests and LASSO regression	Pre-fusion and post-fusion CT radiomics–deep learning model	Random 7:3 training/testing split	Fusion model AUC 0.865; radiomics model AUC 0.803; deep-learning model AUC 0.830; clinical-imaging model AUC 0.745	Retrospective single-study cohort; early-access article with final volume/article number not yet visible	Early adenocarcinoma-spectrum GGN infiltration fusion study
Hu et al. [76]	CT	513 histopathology-confirmed GGNs from two centres	Benign vs malignant ground-glass nodules / early-stage LUAD	3D U-Net segmentation, transfer-learning DNN, CT radiomics with score-level fusion	Score-level fusion of DL and radiomics outputs	DNN + radiomics + SVM fusion	Independent testing dataset	Fusion model AUC 0.73 *±* 0.06; accuracy 75.6%; F1 84.6%	Limited dataset; class imbalance	Histopathology-confirmed GGN fusion study
Jha et al. [77]	CT	4,834 CT scans from a LIDC-IDRI-derived dataset	Normal vs NSCLC-infected CT classification	Handcrafted, deep and Fractional Fourier Transform features after denoising	Feature extraction after FrFT-based preprocessing	Two-layer lightweight neural network	Benchmark dataset evaluation	Reported accuracy 100%; sensitivity 100%; AUROC 1.00	Very high performance suggests overfitting or dataset-separation risk; external validation unclear	Lightweight handcrafted-feature neural-network reference with overfitting caveat
Kanneboina et al. [78]	CT	IQ-OTH/NCCD CT dataset with malignant, benign and normal cases	Early lung cancer detection / malignant, benign and normal classification	GLCM handcrafted texture features + CNN deep semantic features + automated ROI localisation	Hybrid handcrafted and deep feature fusion	Hybrid GLCM-CNN feature extraction + SVM classifier	Experimental dataset evaluation	Accuracy 99%	Conference paper; dataset-specific performance may overestimate generalisability; external clinical validation not reported	GLCM-CNN hybrid detection study
Khademi et al. [?]	CT	114 pathologically proven subsolid nodules	Predicting invasiveness of subsolid lung nodules	SWin Transformer deep path plus radiomics path fused with cross-attention	Cross-attention feature fusion	SWin Transformer + radiomics cross-attention classifier	Ten-fold cross-validation	Accuracy 89.46%; sensitivity 83.99%; specificity 94.99%	Small in-house dataset; conference paper	Cross-attention transformer+radiomics reference
Khademi et al. [79]	CT	114 pathologically proven LUAD subsolid nodules	Pre-invasive/MIA vs invasive subsolid LUAD nodules	I-VISTA: Swin Transformer + CAE Transformer + 3D radiomics with criss-cross attention fusion	Criss-cross attention-based fusion	Triple-path transformer + radiomics fusion classifier	Ten-fold cross-validation	Accuracy 93.93%; sensitivity 92.66%; specificity 94.99%; AUC 0.93	Small in-house dataset; needs external validation	Recent triple-path transformer+radiomics study
Lan et al. [80]	Low-dose CT	322 patients with surgically resected stage Ia LUAD from two centres	Predicting postoperative recurrence of stage Ia lung adenocarcinoma	LDCT radiomics features + ResNet50 deep-learning features + clinical variables	ICC reproducibility assessment and LASSO-based feature selection	Deep-learning radiomics nomogram using ResNet50 and logistic-regression radiomics model	Training, internal validation and external validation cohorts	Combined model AUC 0.972 training, 0.925 internal validation and 0.915 external validation	Retrospective two-centre design; recurrence prediction rather than diagnostic classification	LDCT-aligned deep-learning radiomics nomogram for early-stage LUAD recurrence prediction
Li et al. [81]	LDCT	431 malignant and 795 benign nodules from LIDC/IDRI	Predicting lung nodule malignancy	3D CNN features from AlexNet/VGG-16/Multi-crop networks fused with handcrafted intensity, geometric and texture features	Sequential forward feature selection on fused CNN + handcrafted features	3D CNN features + handcrafted features + SVM	Benchmark evaluation on LIDC/IDRI	Proposed fusion achieved the highest AUC, accuracy, sensitivity and specificity among compared models	Public dataset; retrospective design; labels are LIDC/IDRI-derived	Foundational 3D CNN + handcrafted feature-fusion reference
Li et al. [82]	LDCT	100 breast cancer patients with lung lesions	Discriminating breast cancer lung metastasis vs primary lung cancer	ResNet18-based multi-input residual CNN features + intra- and peri-tumoral radiomics features	Multi-region feature fusion from intra-tumoral and peri-tumoral regions	ResNet18 + radiomics fusion classifier	Five-fold cross-validation	Fusion model AUC 0.913	Includes breast cancer metastasis cohort; not purely early lung cancer detection	Intra/peritumoral DL+radiomics reference for primary-vs-metastatic lung lesion classification
Liang et al. [83]	CT	Mixed NSCLC-Radiomics and NSCLC-Radiogenomics CT dataset	Classifying NSCLC histological subtypes: adenocarcinoma vs squamous-cell carcinoma	Radiomics features + 3D CNN deep features	Multi-head attention feature integration	Radiomics + 3D CNN model with multi-head attention classifier	Model evaluation on mixed public NSCLC datasets	Accuracy 0.88; AUC 0.89	Public-dataset study; external clinical validation not clearly reported	Multi-head attention subtype-classification study
Lin et al. [84]	CT	915 patients with solitary solid pulmonary nodules and suspicious CT signs	Differentiating granuloma nodules from solid lung cancer nodules	3D intranodular, perinodular and gross nodular radiomics features + clinical features	mRMR and XGBoost feature selection	Clinical-image-radiomics deep-learning model (CIRDL)	Five-fold cross-validation	Best CIRDL model AUC 0.9069	Retrospective cohort; differential-diagnosis task rather than screening	Strong 3D deep-radiomics differential-diagnosis reference
Liu et al. [85]	CT	Public and private NSCLC CT datasets	NSCLC subtype classification	Radiomics and deep-learning features with causal-representation learning	Hybrid representative causal feature modelling	Hybrid Representative Causal Network (HRCL)	Public and private dataset evaluation	Accuracy 83.7% on public NSCLC dataset and 90.3% on private NSCLC dataset	Needs further clinical validation; causal assumptions require careful interpretation	Causal-fusion NSCLC subtype study
Zhang et al. [86]	CT	317 histologically confirmed NSCLC patients from TCIA	Non-invasive prediction of NSCLC pathological subtypes	1834 CT radiomic features	LASSO feature selection	Eleven machine-learning classifiers including RF, XGBoost, ET, GB and SVM	Cross-validation and independent testing	Cross-validation AUCs up to 1.000; independent testing AUCs 0.644 for LUAD, 0.618 for LUSC and 0.638 for LCC	External/generalisation performance modest despite high cross-validation results	Verified CT radiomics subtype-classification study
Oh et al. [87]	CT	235 internal NSCLC patients + NSCLC-Radiomics external validation cohort	Classifying NSCLC adenocarcinoma vs squamous-cell carcinoma	Radiomics features + deep-learning features from CT images	PCA and $\ell_{2,1}$-norm minimisation for feature reduction and selection	Radiomics + deep-feature hybrid classifier	Internal test set + external validation on NSCLC-Radiomics	Combined features average AUC 0.7209 and accuracy 0.6564	Moderate performance; subtype classification rather than detection	Cross-cohort subtype DL+radiomics study
Paul et al. [88]	LDCT / CT	National Lung Screening Trial subset	Predicting nodule malignancy in lung screening	CNNs, pre-trained CNN and radiomics features with ensemble classifier	Ensemble feature aggregation	CNN ensemble + radiomics	Unseen test set	Best AUC 0.96; accuracy 89.45%	Conference paper; dataset subset reproducibility needs checking	Early NLST DL+radiomics ensemble reference
Paul et al. [89]	LDCT	NLST subset of nodule-positive and screen-detected lung cancer cases	Nodule malignancy prediction	CNN trained on Law’s 3D texture feature images from nodules	Details extracted from the full-text methods section	CNN ensemble on texture feature images	Model evaluation	Accuracy 79.32%; AUC 0.88	Conference paper; moderate performance	Texture-image deep radiomics reference
Peng et al. [90]	LDCT	415 pathology-confirmed LDCT patients from two centres	Malignancy classification of pulmonary nodules	Voxel normalisation, FFT-based contour fusion, ROI radiomics and patch-based deep learning	Texture-feature selection and interpretable contour fusion	ROI-based machine learning and patch-based deep-learning models	Two-centre evaluation	ROI ML accuracy 92.6%; patch-based DL accuracy 98.5%	Focus partly on preprocessing/normalisation; external prospective validation still needed	Recent two-centre LDCT study on voxel normalisation, contour fusion and malignancy classification
Santos et al. [91]	FDG PET/CT	113 participants with indeterminate solid pulmonary nodules assessed using 2-[18F]FDG PET images	Benign versus malignant pulmonary nodule classification	3D PET image features learned directly by CNN models	End-to-end 3D deep learning with original and augmented datasets; transfer learning using ImageNet-pretrained ResNet-50 was also evaluated	Stacked 3D CNN, VGG-like, Inception-v2-like, and transfer-learning CNN models, with the final model selected as a Stacked 3D CNN	Held-out test-set evaluation after four-fold cross-validation for candidate model selection; Grad-CAM was used for interpretability	The final Stacked 3D CNN achieved test AUC 0.8385, sensitivity 80.00%, specificity 69.23%, and accuracy 73.91%	Retrospective PET-image study with a modest sample size; performance uncertainty was reflected by a wide 95% CI for AUC, and external validation was not reported	Relevant PET-based 3D CNN benchmark for benign–malignant pulmonary nodule classification with Grad-CAM interpretability
Saihood et al. [92]	CT	LIDC-IDRI volumetric nodule dataset	Lung nodule classification	Multi-orientation local texture features and guided attention fusion	Texture feature descriptors + attention-based fusion	Multi-orientation guided-attention deep classifier	Experimental evaluation	Improved classification accuracy over baselines on LIDC-IDRI	Public dataset; computational complexity	Guided-attention texture+DL reference
Shi et al. [93]	CT	Multicentre retrospective LUAD cohort	Three-class LUAD subtyping: AIS, MIA and invasive adenocarcinoma	Central-slice CT anatomy + MRF-mapped radiomics dynamics	MRF-derived dynamic radiomics representation	Dynamic radiomics fusion network	Training, internal testing and external validation cohorts	External validation AUC 0.925; F1-score 0.878; class AUCs reported as MIA 0.97, AIS 0.99 and IAC 0.95	Recent 2026 article; full methodological appraisal still needed	Strong recent dynamic-fusion study for LUAD subtyping
Shivwanshi and Nirala [94]	CT	Lung cancer CT image dataset	Lung nodule multiclass malignancy classification	Hybrid feature-fusion approach combining handcrafted radiomic features, InceptionNet CNN features, and Vision Transformer features	XGBoost-based feature selection applied after handcrafted radiomic, CNN, and Vision Transformer feature extraction	IncCat-LCC: Explainer framework with GPU-based CatBoost classifier and XAI-based model interpretation	Model evaluation using multiclass classification metrics and explainable AI analysis	The proposed framework achieved accuracy 96.74%, precision 93.68%, recall 96.74%, F1-score 95.19%, specificity 98.47%, and AUC 99.76%	Details extracted from the full-text methods section	XAI-enabled handcrafted radiomics, CNN, and Vision Transformer feature-fusion study for lung cancer malignancy classification
Ma et al. [95]	CT	LIDC-IDRI dataset	Benign vs malignant lung nodule classification	Radiomics features fused with multiple deep CNN and graph convolutional network features	Radiomics, GCN-based feature aggregation and ensemble decision fusion	RGD fusion algorithm combining radiomics, GCN and multiple deep CNNs	10-fold cross-validation on LIDC-IDRI	Accuracy 93.25%; sensitivity 89.22%; specificity 95.82%; precision 92.46%; F1-score 0.9114; AUC 0.9629	Public-dataset study; external clinical validation not reported	Strong verified radiomics–deep-learning–graph fusion
Singh et al. [96]	PET/CT + CT	NSCLC Radiogenomics and Radiomics datasets	Early lung cancer detection and therapy-response prediction	PET 3D-CNN features + CT Vision Transformer features with cross-attention fusion	Cross-attention adaptive feature alignment	Multimodal Transformer Fusion model	Conference model evaluation	AUC 0.935; accuracy 92.6%; sensitivity 90.4%	Conference paper; mixes detection and therapy-response prediction; validation details require full text	PET/CT cross-attention transformer fusion reference
Sun et al. [97]	CT	252 pathologically confirmed GGNs; 177 training and 75 testing	Differentiating MIA vs invasive adenocarcinoma in GGNs	Radiomics, 2D deep-learning and 3D deep-learning features with early and late fusion strategies	Pearson correlation, ICC filtering, PCA and LASSO feature selection	SVM-based radiomics/DL classifiers with early and late fusion	Training and testing cohorts	Late fusion test AUC 0.898; early fusion 0.857; 3D DL 0.847; radiomics 0.794; 2D DL 0.754	Retrospective single-centre design; larger multicentre validation needed	Early-vs-late fusion benchmark for GGN invasiveness prediction
Torres et al. [98]	CT	Screening-oriented CT cohort of 60 recruited patients with pulmonary nodules; model optimisation used 51 nodules	Benign versus malignant lung nodule classification in lung cancer screening	2D radiomic embedding based on PyRadiomics texture descriptors from masked nodule slices, including GLCM-derived features	Statistical-significance-based selection of radiomic features correlated with malignancy, followed by low-dimensional radiomic embedding	Optimised feedforward neural network using radiomic embedding	Model optimisation with an independent patient test set, including cases from the local database and LIDC-IDRI	The best model achieved 100% sensitivity, 83% specificity, and AUC 0.94 for malignancy detection on an independent set of patients	Relatively small cohort; external generalisability remains limited despite independent-set evaluation	Screening-aligned radiomics-embedding neural-network study for reducing false positives in CT-based lung cancer screening
Xia et al. [99]	CT	373 pathologically confirmed GGNs from 323 stage-I LUAD patients across two centres	Predicting invasiveness risk of stage-I LUAD manifesting as GGNs	Deep-learning features and radiomics features from CT images	Separate DL and radiomics feature modelling followed by score-level fusion	DL model, radiomics model and fusion classifier	Two-centre evaluation with independent testing/observer comparison	Fusion AUC 0.90 *±* 0.03; radiomics AUC 0.87 *±* 0.04; DL AUC 0.83 *±* 0.05; model accuracy 80.3%	Retrospective design; limited external generalisability	Early-stage LUAD GGN deep-learning–radiomics fusion study
Yang et al. [100]	CT	Pulmonary-RadPath dataset containing 5,134 pathologically confirmed pulmonary lesion CT images	Hierarchical classification of pulmonary lesions, including invasive and non-invasive adenocarcinoma, squamous carcinoma, tuberculosis, and hamartoma	Deep-learning representation learning from CT images using pathology-confirmed hierarchical labels	Hierarchical label modelling to address label ambiguity rather than handcrafted feature selection	Leaky Dense Hierarchy deep-learning classification model	Large-scale retrospective radio-pathomics evaluation using pathologically confirmed pulmonary-lesion labels	The proposed Leaky Dense Hierarchy approach outperformed prior methods in hierarchical pulmonary-lesion classification, with advantages in dataset scale, disease coverage, and hierarchical label modelling	Retrospective dataset-based study; not specifically a radiomics-feature fusion model, and prospective external clinical validation was not reported	Relevant differential-diagnosis study for lung cancer-related pulmonary lesions using pathology-confirmed CT labels and hierarchical deep learning
Ma et al. [95]	CT	LIDC-IDRI dataset	Benign vs malignant lung nodule classification	Radiomics features fused with graph convolutional network features and multiple deep CNN features	GCN-based feature aggregation with ensemble decision fusion	RGD fusion algorithm combining radiomics, GCN and multiple deep CNNs	10-fold cross-validation on LIDC-IDRI	Accuracy 93.25%; sensitivity 89.22%; specificity 95.82%; precision 92.46%; F1-score 0.9114; AUC 0.9629	Public-dataset study; external clinical validation not reported	Strong verified graph-based radiomics–deep-learning fusion
Zhang and Meng [101]	CT	LIDC-IDRI dataset	Pulmonary nodule classification	Improved random-walk lung parenchyma segmentation; VLDTP texture features + gray histogram features + VGG16 deep features	Multi-feature fusion in VGG16 classification framework	VGG16 + handcrafted feature-fusion classifier	Experimental validation on LIDC-IDRI	VGG16 with explainable handcrafted features improved classification accuracy by 0.045	Public-dataset study; exact final metrics should be checked from full text	VGG16 + handcrafted feature-fusion reference
Zhao et al. [102]	CT	449 clinical stage IA lung adenocarcinoma patients from two hospitals	Predicting visceral pleural invasion in clinical stage IA lung adenocarcinoma	Radiomics, deep-learning and fusion features	Feature-level and decision-level fusion	Late-fusion deep learning radiomics model	Training cohort and external test cohort	Late-fusion model external AUC 0.812	Predicts VPI rather than benign/malignant diagnosis or subtype; retrospective design	Relevant deep-radiomics fusion study for early LUAD risk stratification

The fusion evidence suggests that attention-based integration is increasingly being used to improve the interaction between deep features and radiomic descriptors. Attention-integrated DeepCNN models combined with reproducible CT radiomic features have been applied to lung cancer subtype classification, with the aim of improving both classification performance and feature robustness across imaging conditions [67]. This direction is important because attention mechanisms may help models focus on diagnostically relevant tumour regions while reducing the influence of irrelevant image areas.

Other fusion studies combined three-dimensional CNNs with handcrafted texture and shape descriptors such as HOG and LBP features. These approaches aimed to integrate deep spatial representations with engineered descriptors of morphology and local texture, and were evaluated across public CT datasets such as IQ-OTH/NCCD, LIDC-IDRI, and NSCLC-Radiomics [68]. Although high performance was reported, such results should be interpreted cautiously because public benchmark datasets may introduce overlap, limited clinical heterogeneity, or incomplete reporting of independent validation.

Fusion was also used for pulmonary nodule malignancy grading. CNN-derived features were combined with radiomics and evolutionary ensemble learning to classify CT signs and malignancy using LIDC-IDRI samples [69]. This type of model is relevant because it links deep feature extraction with radiomics-based nodule characterisation. However, LIDC-IDRI malignancy labels may include radiologist-derived scores rather than uniformly pathology-confirmed outcomes, which limits certainty about clinical translation.

Weighted multi-stream fusion was another strategy identified in the fusion evidence base. Three-stream models combined residual deep neural network features, custom deep neural network features, and handcrafted frequency-domain features before classification using an SVM [70]. Such designs provide a clear example of feature- or score-level integration between deep and handcrafted representations. However, several studies in this group did not fully report dataset size, independent validation, or external reproducibility, limiting their contribution to generalisable evidence.

Overall, DL–radiomics fusion studies suggest that combining handcrafted and deep image features can improve representation of lung lesions for subtype classification, malignancy grading, and lung cancer classification. The strongest methodological value of this category is that fusion models attempt to balance interpretability and predictive power: radiomics features provide explainable quantitative descriptors, while deep learning features capture complex spatial patterns that may be difficult to predefine manually. However, the fusion evidence remains heterogeneous. Several studies did not clearly specify whether fusion occurred at feature level, decision level, or model level, and many had incomplete reporting of dataset size, validation design, or external testing. These limitations justify the need for a formal fusion taxonomy and careful comparison against radiomics-only and deep learning-only baselines.

### Taxonomy and mathematical formalisation of fusion strategies

Fusion strategies were reported inconsistently across the included studies, this review formalised fusion models into three main categories: early fusion, late fusion, and hybrid or intermediate fusion. This taxonomy was informed by multimodal machine-learning and medical data-fusion literature, where fusion is commonly distinguished according to whether integration occurs at the feature, representation, or decision level [103–105]. The taxonomy was used to distinguish whether radiomics, deep learning, and clinical or semantic variables were combined before modelling, after independent model prediction, or through multi-stage feature and decision integration.

Let *R* represent handcrafted radiomics features, *D* represent deep learning-derived features, and *C* represent clinical, semantic radiological, or other non-imaging variables. In early fusion, all available features are concatenated before being passed into a single classifier, as shown in Eq. (1):

1 $$\hat{y} = f([R, D, C])$$

where *f(·)* represents a machine-learning or deep-learning classifier and *[R,D,C]* denotes feature-level concatenation. As expressed in Eq. (1), this approach was common in studies that combined radiomics features, deep features, and clinical variables into a single feature vector before classification [104, 105].

In late fusion, separate models are trained on different feature sources and their outputs are combined at the decision level, as formalised in Eq. (2):

2 $$\hat{y} = g(f_R(R), f_D(D), f_C(C))$$

where $f_R(R)$, $f_D(D)$, and $f_C(C)$ represent modality- or feature-specific prediction functions, and *g(·)* represents the decision-level fusion rule, such as weighted averaging, voting, stacking, or ensemble learning. As shown in Eq. (2), late fusion is useful when different feature sources have distinct predictive roles or when independent models are easier to interpret [103, 104, 106].

Hybrid or intermediate fusion combines elements of both early and late fusion. In this setting, selected feature representations are first transformed or embedded, then combined with other variables before final classification, as defined in Eq. (3):

3 $$\hat{y} = g(h([\phi_R(R), \phi_D(D)]), C)$$

where $\phi_R(R)$ and $\phi_D(D)$ represent transformed radiomics and deep feature embeddings, *h(·)* represents an intermediate fusion layer or representation-learning function, and *g(·)* represents the final prediction function. Equation (3) captures hybrid fusion approaches observed in studies using attention mechanisms, multi-stream architectures, ensemble learning, or multi-stage classifiers [67, 70, 103, 105].

This taxonomy, formalised in Eq. (1)–(3), helps clarify the theoretical boundaries between fusion strategies. Early fusion emphasises feature-level integration, late fusion emphasises decision-level integration, and hybrid fusion combines representation-level and decision-level learning. However, many studies described their approach only as “combined”, “integrated”, or “fusion” without specifying the exact level of integration. This inconsistency limited direct comparison across studies and contributed to methodological heterogeneity [67, 70, 104, 105].

### Comparative synthesis across model categories

Across the included detection and classification studies, radiomics-only, deep learning-only, and DL–radiomics fusion models showed different methodological strengths and limitations. Radiomics-only models were generally more interpretable because their features described predefined image characteristics such as shape, texture, intensity, wavelet patterns, and lesion heterogeneity. However, these models were sensitive to segmentation methods, scanner protocols, image reconstruction parameters, and feature-selection pipelines.

Deep learning-only models achieved strong reported performance in several studies, particularly for automated nodule detection and CT-based classification. Their main advantage was automated feature learning from image data, reducing dependence on handcrafted feature engineering. However, DL-only models were often limited by black-box behaviour, possible overfitting, unclear data splits, public dataset overlap, and limited prospective or external validation.

DL–radiomics fusion models attempted to combine the strengths of both approaches. By integrating handcrafted radiomics with deep learned features, these models aimed to improve lesion representation while retaining some interpretability. Fusion models frequently reported high internal performance, and some studies demonstrated stronger generalisability when external validation was included. Nevertheless, fusion studies were also the most heterogeneous, with variation in fusion level, feature dimensionality, classifier choice, modality, sample size, and validation strategy.

Overall, the evidence suggests that fusion models may offer added value over radiomics-only or DL-only baselines, particularly when they are externally validated and clearly define the level of feature integration. However,as many studies used different datasets, outcome definitions, imaging protocols, and validation approaches, direct performance ranking should be interpreted cautiously.

### Sources of heterogeneity across included studies

Substantial heterogeneity was observed across the included detection and classification studies. This heterogeneity limited the appropriateness of formal statistical pooling and required a structured narrative and quantitative synthesis by model category. The main sources of heterogeneity were imaging modality, dataset design, sample size, outcome definition, segmentation method, feature extraction pipeline, model architecture, fusion strategy, validation approach, and reporting completeness [17, 107, 108].

First, imaging heterogeneity was evident across CT, LDCT, spectral CT, and PET/CT studies. Differences in acquisition protocol, slice thickness, reconstruction kernel, contrast use, scanner manufacturer, and image preprocessing can influence both radiomics features and deep learning representations [29, 40, 109]. This was particularly important for radiomics-only models, where handcrafted features are sensitive to voxel spacing, intensity discretisation, reconstruction parameters, and segmentation boundaries [5, 29, 40].

Second, dataset heterogeneity was substantial. Some studies used public benchmark datasets such as LIDC-IDRI, LUNA16, NLST, NSCLC-Radiomics, or IQ-OTH/NCCD, whereas others used single-centre or multicentre institutional cohorts [36, 62, 71]. Public datasets improved comparability but often relied on radiologist-derived labels or benchmark-specific preprocessing. In contrast, institutional datasets sometimes used pathology-confirmed outcomes but were frequently smaller, retrospective, and less externally validated [23, 31, 49].

Third, outcome heterogeneity limited direct comparison across studies. Although all included studies were relevant to detection or classification, their endpoints varied widely, including benign–malignant nodule classification, lung cancer versus non-cancer classification, ground-glass nodule invasiveness prediction, histological subtype classification, malignancy grading, and false-positive reduction [22, 31, 45]. These outcomes are clinically related but not statistically interchangeable, making pooled performance estimates difficult to justify.

Fourth, validation heterogeneity was a major limitation. Some studies used simple training and testing splits, others used k-fold cross-validation, and a smaller number used independent or external validation cohorts [23, 26, 71, 73]. Multicentre validation was uncommon but provided stronger evidence of generalisability [49, 71, 73]. Studies reporting only internal validation were more vulnerable to overfitting, dataset-specific bias, and optimistic performance estimates [107, 110].

Fifth, model-level heterogeneity was particularly evident among fusion studies. Some models used feature-level concatenation, others used weighted decision fusion, stacking ensembles, attention-based fusion, or multi-stage representation learning [68–71]. However, several studies did not clearly define the level at which fusion occurred. This inconsistency made it difficult to determine whether reported performance gains were attributable to the fusion strategy itself or to confounding factors such as dataset size, classifier choice, feature dimensionality, segmentation quality, or validation design [71, 73, 107].

Taken together, these sources of heterogeneity explain why formal meta-analysis was not undertaken. Instead, this review used structured subgroup comparison across radiomics-only, deep learning-only, and DL–radiomics fusion models, with attention to validation design, modality, task definition, and generalisability. This approach allowed meaningful comparison while avoiding statistically inappropriate pooling of non-equivalent diagnostic tasks and model designs [14, 107, 111].

### Methodological implications for generalisable fusion models

The findings from the structured synthesis have several implications for the development of generalisable deep learning–radiomics fusion models for early lung cancer detection as components of clinical decision support systems. First, future fusion models should be designed around clearly defined clinical tasks. The included studies used heterogeneous endpoints, including benign–malignant pulmonary nodule classification, lung cancer detection, ground-glass nodule invasiveness prediction, histological subtype classification, and malignancy grading. Although these tasks are related, they require different reference standards, model outputs, and validation designs. A proposed methodological framework should therefore define the target outcome explicitly before model development.

Second, the evidence suggests that fusion models should not simply combine features for performance optimisation without explaining the level of integration. Several studies reported “combined” or “integrated” models without specifying whether fusion occurred at feature level, decision level, model level, or through an intermediate attention or representation-learning layer. This lack of clarity limits reproducibility and makes it difficult to compare fusion strategies across studies. A proposed methodological framework should therefore report the fusion mechanism explicitly, including whether radiomics features, deep learning embeddings, and clinical variables are concatenated before classification, combined after separate model prediction, or integrated through a hybrid architecture.

Third, external validation should be treated as a central design requirement rather than an optional evaluation step. Many studies reported strong internal performance but lacked independent testing across centres, scanners, reconstruction protocols, or patient populations. This is particularly important for radiomics and deep learning because both approaches can be sensitive to imaging protocol variation and dataset-specific bias. Generalisable fusion models should therefore include site-held-out validation, multicentre testing, or external validation cohorts whenever possible.

Fourth, harmonisation and standardisation are necessary for robust multi-centre performance. Radiomics features are affected by segmentation, voxel spacing, discretisation, reconstruction kernel, and preprocessing choices, while deep learning models may learn scanner-specific or centre-specific patterns. A proposed methodological framework should therefore include standardised preprocessing, transparent segmentation protocols, radiomics feature harmonisation, and sensitivity analysis across acquisition settings.

Fifth, interpretability should be integrated into model development. Radiomics-only models offer some interpretability because their features correspond to predefined quantitative image descriptors, whereas deep learning-only models are often more difficult to explain. Fusion models provide an opportunity to combine predictive performance with interpretability, but this requires transparent reporting of selected radiomics features, visual explanation of deep learning attention or saliency, and clinical interpretation of model outputs. Explainability methods should therefore be used not only for visualisation but also for checking whether models rely on clinically meaningful tumour or nodule regions, which is essential for CDSS adoption.

Finally, the synthesis indicates that future fusion models should report performance beyond AUC or accuracy. Sensitivity, specificity, precision, F1-score, calibration, confidence intervals, decision-curve analysis, and external validation performance are needed to evaluate clinical usefulness. Without these metrics, it remains difficult to determine whether a model is suitable for early detection, triage, or decision support. A proposed methodological framework should therefore combine diagnostic performance reporting with calibration, robustness testing, and transparent validation.

### Identified gaps in the literature

Despite considerable progress in applying radiomics, deep learning, and DL–radiomics fusion models to early lung cancer detection and classification, the revised synthesis identified several persistent methodological gaps that continue to limit clinical translation, reproducibility, and generalisability. These gaps were observed across radiomics-only, deep learning-only, and fusion-based studies, particularly in relation to external validation, harmonisation, fusion reporting, outcome definition, and clinical interpretability.

#### Limited multi-centre validation and domain generalisation

A major limitation across the reviewed literature was the limited use of robust multi-centre validation. Many studies relied on single-centre retrospective datasets, internal cross-validation, random train–test splits, or public benchmark datasets. Although these designs are useful for early model development, they provide limited evidence of real-world generalisability across hospitals, scanners, reconstruction kernels, patient populations, and screening settings.

This limitation was evident across all three model categories. Radiomics-only models were particularly affected by acquisition and reconstruction variability because handcrafted features are sensitive to voxel spacing, intensity discretisation, segmentation boundaries, and reconstruction parameters. Deep learning-only models also showed vulnerability to domain shift, especially where high performance was reported on public datasets without clearly independent external testing. Fusion models were expected to improve robustness by combining complementary feature sources, but several studies still lacked site-held-out validation or multicentre testing.

Only a smaller subset of studies included external validation cohorts or cross-cohort evaluation. These studies were especially valuable because they demonstrated that strong internal performance does not always translate into stable external performance. This reinforces the need for future models to incorporate multicentre validation, scanner-level robustness testing, and site-held-out evaluation as standard components of model development.

#### Inconsistent feature harmonisation and reproducibility practices

Reproducibility safeguards were inconsistently implemented across radiomics and fusion pipelines. Some studies used standardised radiomics extraction tools, feature-selection procedures, or harmonisation approaches, but many did not fully report preprocessing parameters, segmentation methods, image resampling, intensity discretisation, or reconstruction settings. This makes it difficult to reproduce extracted features or compare radiomics signatures across cohorts.

Radiomics-only models were especially affected by this gap because their performance depends heavily on the stability of handcrafted features. Inconsistent segmentation, heterogeneous CT acquisition protocols, and scanner-dependent variation can alter feature values and reduce model transferability. Fusion models inherit these radiomics-related limitations while adding further complexity through deep feature extraction, multimodal integration, and classifier selection.

Future studies should therefore report radiomics pipelines in sufficient detail, including image preprocessing, segmentation strategy, feature classes, feature-selection methods, and harmonisation procedures. Where multicentre data are used, harmonisation methods such as ComBat-style adjustment or site-aware modelling should be considered. Feature stability testing, inter-observer segmentation analysis, and sensitivity analysis across reconstruction protocols should also be included where possible.

#### Unclear reporting of fusion strategies

A key gap identified in the DL–radiomics fusion literature was the inconsistent reporting of fusion strategy. Several studies used terms such as “combined”, “integrated”, or “fusion” without clearly specifying whether integration occurred at feature level, decision level, model level, or through an intermediate representation-learning layer. This lack of clarity limits reproducibility and makes it difficult to determine whether observed performance improvements are attributable to the fusion strategy itself or to other factors such as feature dimensionality, classifier choice, sample size, or validation design.

To address this gap, fusion studies should explicitly define the input feature sources, the stage at which integration occurs, and the classifier or learning architecture used after fusion. Studies should also compare fusion models against radiomics-only and deep learning-only baselines using the same dataset, outcome definition, and validation strategy. Without such controlled comparisons, claims of fusion superiority remain difficult to interpret.

#### Heterogeneous outcome definitions

Another important gap was the heterogeneity of outcome definitions. The included studies addressed clinically related but distinct tasks, such as lung cancer detection, benign–malignant pulmonary nodule classification, lung cancer versus non-cancer classification, histological subtype classification, ground-glass nodule invasiveness prediction, malignancy grading, and false-positive reduction. These outcomes are not statistically interchangeable and should not be pooled without careful consideration.

This heterogeneity complicates comparison across studies because a model designed for benign–malignant nodule classification is not directly equivalent to one designed for subtype classification or invasiveness prediction. Future studies should define the clinical task precisely, state the intended use case, and report the reference standard clearly. This is particularly important for early detection, where screening, triage, diagnosis, and pathological risk prediction have different clinical implications.

#### Incomplete performance reporting and limited calibration analysis

Many studies reported AUC or accuracy but did not consistently report sensitivity, specificity, precision, F1-score, confidence intervals, calibration, or decision-curve analysis. This limits clinical interpretation because early lung cancer detection requires careful balancing of false positives and false negatives. High AUC alone does not indicate whether a model is clinically useful, calibrated, or suitable for screening deployment.

Future studies should report a broader set of diagnostic metrics and include calibration assessment where possible. Calibration is particularly important for risk prediction models because poorly calibrated models may overestimate or underestimate malignancy probability. Decision-curve analysis may also help determine whether model predictions provide clinical benefit across relevant risk thresholds.

#### Limited explainability and clinical interpretability

Although radiomics models provide some interpretability through handcrafted features, many studies did not provide sufficient clinical interpretation of selected features. Deep learning-only models were often limited by black-box behaviour, and fusion models added further complexity by combining multiple feature sources. Explainability methods such as Grad-CAM, saliency maps, attention maps, feature-importance plots, SHAP analysis, or radiomics feature interpretation were inconsistently applied.

Future fusion models should integrate explainability into model development and evaluation. Explainability should not be limited to visual examples; it should help determine whether models rely on clinically meaningful tumour or nodule regions and whether selected radiomics or deep features align with known imaging phenotypes. This is essential for clinical trust, regulatory acceptance, and translation into CDSS workflows.

#### Limited prospective and clinical workflow evaluation

Most studies remained retrospective and algorithm-focused. Few evaluated how models would perform in real clinical workflows, screening pathways, multidisciplinary decision-making, or radiologist-AI collaboration. As a result, the clinical utility of many models remains uncertain despite strong reported performance.

Future studies should move beyond retrospective model development toward prospective validation, workflow integration, and evaluation of clinical impact. This includes assessing whether models reduce false positives, improve early detection, support radiologist decision-making, or improve triage of indeterminate pulmonary nodules. Without workflow-level evaluation, the translation of DL–radiomics fusion models into clinical practice will remain limited.

## Proposed methodological framework for generalisable DL–radiomics fusion as a clinical decision support system

**Fig. 2:**
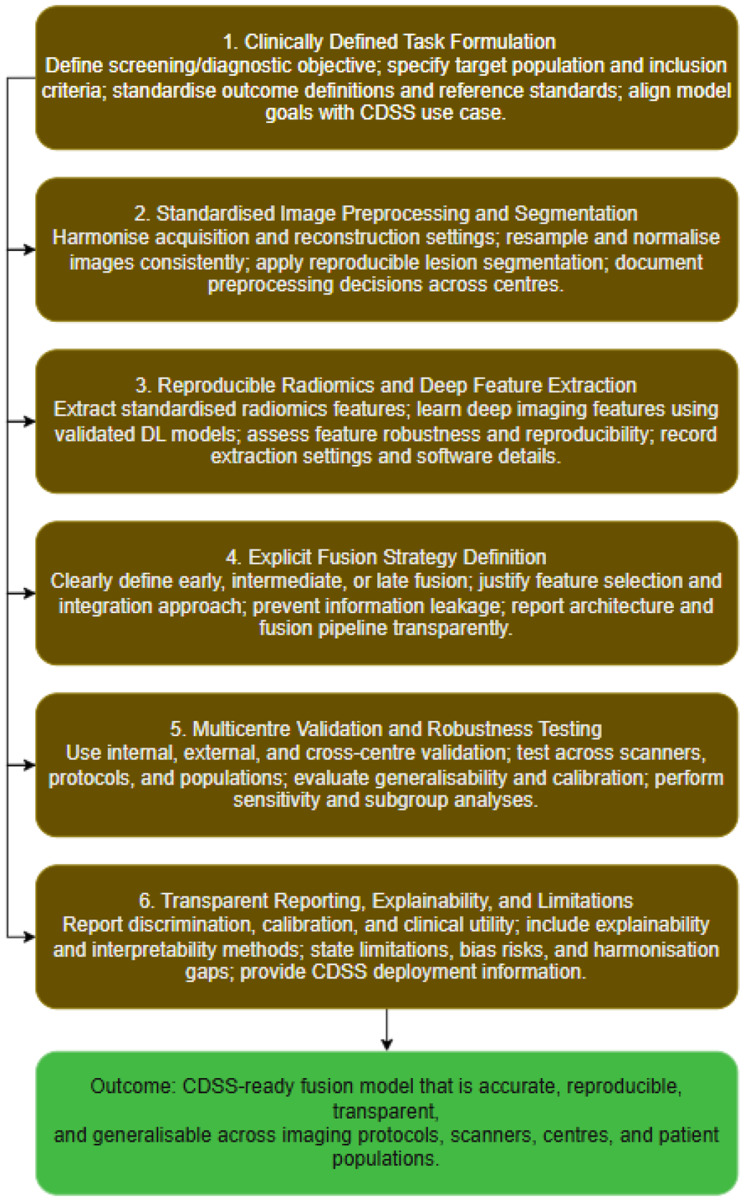
Proposed methodological framework for developing generalisable DL–radiomics fusion models as clinical decision support systems for early lung cancer detection

Based on the structured synthesis, this section presents a proposed methodological framework for developing generalisable deep learning–radiomics fusion models for early lung cancer detection Fig. 2, intended for deployment as components of a clinical decision support system (CDSS). The proposed methodological framework is designed to address the main weaknesses identified across the included studies, including heterogeneous outcome definitions, inconsistent fusion reporting, limited external validation, incomplete harmonisation, and variable performance reporting. Rather than presenting a generic checklist, the proposed methodological framework links each methodological stage to specific limitations observed in radiomics-only, deep learning-only, and DL–radiomics fusion studies, and aligns each stage with the practical requirements of CDSS deployment in screening and diagnostic workflows.

The proposed methodological framework consists of six sequential components: clinically defined task formulation, standardised image preprocessing and segmentation, reproducible radiomics and deep feature extraction, explicit fusion strategy definition, multicentre validation and robustness testing, and transparent reporting of performance, calibration, explainability, and limitations. These components are intended to support the development of CDSS-ready fusion models that are not only accurate in internal datasets, but also reproducible and generalisable across imaging protocols, scanners, centres, and patient populations.

### Clinically defined task formulation

The synthesis showed that included studies used different but related endpoints, including benign–malignant pulmonary nodule classification [26], lung cancer versus non-cancer classification, ground-glass nodule invasiveness prediction [24], histological subtype classification [83], malignancy grading, and false-positive reduction. These outcomes cannot be treated as interchangeable because they differ in clinical purpose, reference standard, class distribution, and model interpretation.

Therefore, future DL–radiomics fusion models intended for CDSS deployment should define the target task before model development. For early lung cancer detection, the task should specify whether the CDSS is intended for screening, triage, diagnostic support, subtype classification, or invasiveness prediction. The target population, imaging modality, inclusion criteria, and reference standard should also be clearly stated. This reduces ambiguity and improves comparability across studies.

### Standardised image preprocessing and segmentation

The second component of the proposed methodological framework is standardised image preprocessing and segmentation. Radiomics features are sensitive to voxel spacing, intensity discretisation, reconstruction kernel, segmentation boundary, and image resampling. Deep learning models may also learn scanner-specific or site-specific patterns if preprocessing is inconsistent. To reduce this source of heterogeneity, images should be processed using transparent and reproducible protocols.

The proposed methodological framework recommends reporting acquisition parameters, reconstruction methods, resampling strategy, intensity normalisation, segmentation method, and quality-control procedures. Where manual or semi-automatic segmentation is used, inter-observer variability should be assessed. Where automatic segmentation is used, segmentation performance should be reported separately before classification performance.

### Reproducible radiomics and deep feature extraction

The third component of the proposed methodological framework is reproducible feature extraction. Radiomics features should be extracted using standardised software and clearly defined feature classes, including first-order, shape, texture, wavelet, histogram, habitat, or peritumoural features where appropriate. Feature selection should be performed only within the training data to avoid information leakage.

For deep learning, the architecture, input size, augmentation strategy, training procedure, hyperparameters, and optimisation method should be reported. If pretrained models are used, the pretraining source should be stated. If deep features are extracted for fusion with radiomics, the layer or embedding used should be clearly defined. This improves reproducibility and allows fair comparison with radiomics-only and DL-only baselines.

### Explicit fusion strategy definition

The fourth component of the proposed methodological framework is explicit fusion strategy definition. The synthesis showed that many studies used terms such as “combined”, “integrated”, or “fusion” without clearly identifying the level of fusion [67, 70]. This limits reproducibility and makes it difficult to determine whether performance gains are caused by the fusion strategy or by other factors such as feature dimensionality, classifier choice, or dataset size.

Future studies should state whether fusion is performed at feature level, decision level, model level, or through a hybrid/intermediate representation. The feature sources should also be defined, including handcrafted radiomics features, deep learning embeddings, clinical variables, semantic radiological features, PET/CT parameters, molecular biomarkers, or pathology features. Fusion models should be compared against radiomics-only and DL-only baselines using the same dataset and validation strategy.

### Multicentre validation and robustness testing

The fifth component of the proposed methodological framework is multicentre validation and robustness testing. A major limitation across the reviewed literature was the reliance on internal validation, single-centre cohorts, or public benchmark datasets [9]. Although these datasets are useful for development, they do not fully capture clinical variation across scanners, institutions, reconstruction protocols, populations, and disease prevalence.

A CDSS-ready fusion model should therefore include external validation, site-held-out testing, or multicentre validation whenever possible. Robustness should also be assessed across scanner type, reconstruction kernel, slice thickness, segmentation method, and class imbalance. Where external validation is not possible, the limitation should be clearly stated and the model should not be overclaimed as generalisable.

### Transparent reporting, calibration, and explainability

The final component of the proposed methodological framework is transparent reporting of performance, calibration, explainability, and limitations. Many studies reported AUC or accuracy but did not consistently report sensitivity, specificity, precision, F1-score, confidence intervals, calibration, decision-curve analysis, or external validation performance [18, 107, 110]. This limits clinical interpretation, especially for early detection where false negatives and false positives have different consequences.

Future studies should report multiple diagnostic metrics, confidence intervals, calibration plots or calibration statistics, and decision-curve analysis where possible. Explainability should also be integrated into model evaluation. For radiomics, selected features should be interpreted clinically where possible. For deep learning, saliency maps, Grad-CAM, attention maps, or similar methods should be used to assess whether the model focuses on relevant lesion regions. For fusion models, explainability should clarify how radiomics, deep features, and clinical variables contribute to predictions, which is essential for clinician trust within a CDSS workflow.

### Framework summary

In summary, the proposed methodological framework recommends that future DL–radiomics fusion studies follow a structured development pathway: define the clinical task, standardise imaging and segmentation, extract reproducible radiomics and deep features, specify the fusion level, validate across independent or multicentre datasets, and report performance transparently with calibration and explainability. This proposed methodological framework directly responds to the main gaps identified in the reviewed literature and provides a more generalisable basis for developing CDSS components for early lung cancer detection.

### Implications of the proposed methodological framework

The proposed methodological framework has important implications for both research and clinical practice, as it offers a structured pathway to move deep learning–radiomics models from single-centre proof-of-concept studies towards robust, multi-centre clinical decision support systems. By explicitly embedding harmonisation, feature reproducibility checks, domain-generalisation strategies, and site-held-out external validation, the proposed methodological framework directly addresses key barriers that have historically limited model transferability, such as scanner heterogeneity, protocol differences, and dataset bias. This should improve the reliability of performance estimates, facilitate fair benchmarking across studies and centres, and make resulting CDSS more acceptable to regulators and guideline bodies that require transparent calibration. In parallel, the integration of explainability methods, simple clinical covariates, and workflow-aware deployment may increase clinician trust and support adoption in early lung cancer screening, staging, and treatment planning. The proposed methodological framework not only synthesises best practice from the current literature but also provides a practical blueprint for future multi-centre studies to design, report, and validate generalisable DL–radiomics fusion CDSS in a way that is reproducible, comparable, and clinically meaningful.

## Limitations of the study and future work

### Limitations of the study

This systematic literature review has several limitations. First, although the search strategy was revised and expanded to include Scopus, PubMed/MEDLINE, IEEE Xplore, and Google Scholar, the final synthesis may still have missed relevant studies because of differences in database indexing, terminology, publication venue, and reporting style. Studies using terms such as computer-aided diagnosis, pulmonary nodule risk prediction, radiogenomics, multimodal imaging, or artificial intelligence-assisted screening may not always use the terms “radiomics”, “deep learning”, or “fusion” consistently. To reduce this risk, database-specific Boolean strings were used; however, complete retrieval of all relevant studies cannot be guaranteed.

Second, the review did not conduct a formal meta-analysis. Although quantitative performance metrics such as AUC, accuracy, sensitivity, specificity, and F1-score were extracted where available, statistical pooling was not considered appropriate because of substantial heterogeneity in imaging modality, dataset design, sample size, segmentation strategy, outcome definition, validation design, model architecture, and fusion strategy. In addition, several studies did not report confidence intervals, standard errors, calibration metrics, or sufficient information required for robust meta-analytic pooling. Therefore, the synthesis was structured by model category rather than pooled effect estimation.

Third, the included literature varied considerably in reporting completeness and methodological quality. Several studies, particularly conference papers and recent AI-focused publications, did not fully report dataset composition, train–test splitting, external validation procedures, segmentation methods, feature-selection steps, calibration, or implementation details. This limited the ability to compare models directly and may have introduced uncertainty into the interpretation of reported performance.

Fourth, the review focused on early lung cancer detection and classification, including benign–malignant pulmonary nodule classification, lung lesion classification, subtype classification, invasiveness prediction, and malignancy prediction. Studies focused primarily on prognosis, recurrence, treatment response, toxicity, EGFR/PD-L1 prediction, lymph-node metastasis, or survival were excluded to maintain alignment with the early-detection objective, which may have excluded some related studies with potentially useful methodological insights.

Fifth, the review relied on reported performance metrics rather than re-analysis of original imaging datasets. As a result, the synthesis could not independently verify model performance, calibration, data leakage, segmentation quality, or robustness across scanner protocols. Reported results, particularly very high accuracy or AUC values from single-centre or public benchmark datasets, should therefore be interpreted cautiously.

Although this review introduced a structured taxonomy of early, late, and hybrid DL–radiomics fusion strategies, the analysis was limited by inconsistent reporting across the included studies. Many studies used broad terms such as “combined model”, “integrated model”, or “fusion model” without clearly specifying whether integration occurred at feature level, decision level, model level, or through intermediate representation learning. This limited the ability to directly compare fusion architectures and determine whether reported performance gains were attributable to the fusion strategy itself or to differences in dataset size, feature dimensionality, classifier choice, validation design, or study population.

Finally, the review was not prospectively registered in PROSPERO or another public protocol registry. Although the review question, eligibility criteria, search strategy, data-extraction variables, and synthesis plan were defined before full-text screening, the absence of prospective registration is acknowledged as a methodological limitation.

### Future work

The limitations identified in this study point directly to several priorities for future work. First, although the search strategy was revised and expanded across Scopus, PubMed/MEDLINE, IEEE Xplore, and Google Scholar, future reviews should broaden the terminology to include related concepts such as computer-aided diagnosis, pulmonary nodule risk prediction, radiogenomics, multimodal imaging, and artificial intelligence-assisted screening, and should consider additional databases such as Web of Science and Embase to improve retrieval completeness. Future reviews should also be prospectively registered in PROSPERO or a similar public protocol registry to strengthen methodological transparency.

Second, future reviews should aim to perform formal meta-analysis where feasible. This will require the primary studies themselves to report confidence intervals, standard errors, confusion matrices, calibration metrics, and complete variance estimates. Authors and journals should therefore encourage standardised reporting of diagnostic performance so that future syntheses can move beyond structured narrative comparison toward quantitative pooling.

Third, future research should improve reporting completeness in primary studies. Authors should fully document dataset composition, train–test splitting, external validation procedures, segmentation methods, feature-selection steps, calibration, and implementation details. Alignment with reporting standards such as PRISMA 2020, TRIPOD+AI, CLAIM, and the Radiomics Quality Score should be a baseline expectation rather than an exception [14, 17, 18, 107].

Fourth, future studies should expand the scope beyond early detection and classification to also examine prognosis, recurrence, treatment response, EGFR/PD-L1 prediction, lymph-node metastasis, and survival within the same DL–radiomics fusion frameworks. This will help determine whether fusion models built for early detection can be extended into a continuum of clinical decision support across the lung cancer care pathway.

Fifth, future research should prioritise prospective and multicentre validation of DL–radiomics fusion CDSS for early lung cancer detection. Studies should use independent site-held-out test cohorts, standardised imaging protocols, transparent segmentation procedures, and harmonised radiomics pipelines. Where possible, models should be evaluated across different scanners, reconstruction kernels, slice thicknesses, and patient populations to assess domain generalisation [8, 9].

Sixth, future studies should re-analyse imaging datasets directly rather than relying solely on reported metrics. This would allow independent verification of model performance, calibration, data leakage, segmentation quality, and robustness across scanner protocols. Open data sharing, federated learning, and reproducible code release should be encouraged to enable this kind of independent validation.

Seventh, future research should define fusion strategies more clearly. Authors should explicitly state whether fusion occurs at feature level, decision level, model level, or through hybrid/intermediate representation learning. Fusion models should be compared against radiomics-only and deep learning-only baselines using the same dataset, preprocessing pipeline, outcome definition, and validation design. This would make it easier to determine whether fusion itself improves performance or whether reported gains arise from differences in model architecture, feature dimensionality, or dataset design [67, 70].

Eighth, future studies should report performance more comprehensively. Beyond AUC and accuracy, studies should include sensitivity, specificity, precision, F1-score, confidence intervals, calibration, decision-curve analysis, and external validation results [18, 107, 110]. Calibration and clinical utility are especially important for early detection because false-positive and false-negative predictions have different consequences for screening, follow-up imaging, biopsy decisions, and patient anxiety.

Finally, future work should move beyond retrospective algorithm development toward clinically integrated evaluation. This includes assessing how DL–radiomics fusion CDSS perform in real screening or diagnostic workflows, whether they improve radiologist decision-making, whether they reduce unnecessary investigations, and whether they support earlier detection of clinically significant lung cancer. Reader studies, prospective workflow evaluation, federated or privacy-preserving learning, and fairness assessment across populations and imaging centres should be prioritised. Greater emphasis should also be placed on explainability, reproducibility, open reporting, and alignment with AI reporting standards so that future CDSS can be translated more safely into clinical practice.

## Conclusion

This review synthesised the revised evidence base on radiomics-only, deep learning-only, and deep learning–radiomics fusion models for early lung cancer detection and classification using CT, LDCT, spectral CT, and PET/CT imaging, framed within the broader objective of developing CDSS for early lung cancer detection. The findings show that radiomics-only models remain valuable because they provide interpretable handcrafted descriptors of lesion shape, intensity, texture, heterogeneity, and peritumoural characteristics. However, their performance is frequently limited by segmentation variability, reconstruction differences, scanner dependence, and incomplete external validation. Deep learning-only models demonstrated strong potential for automated nodule detection and lung cancer classification, particularly when three-dimensional CNNs, residual networks, capsule networks, contrastive learning, or transformer-based architectures were used. Nevertheless, these models were often constrained by black-box behaviour, unclear data splitting, public dataset overlap, and limited evidence of prospective or multicentre generalisability.

Deep learning–radiomics fusion models appeared to offer the most promising direction because they combine the interpretability of handcrafted radiomics with the representational strength of deep learning. Fusion approaches were particularly relevant for benign–malignant pulmonary nodule classification, lung cancer subtype classification, ground-glass nodule invasiveness prediction, and malignancy grading. However, the evidence remains methodologically heterogeneous. Studies varied substantially in imaging modality, segmentation approach, feature extraction pipeline, classifier choice, fusion strategy, outcome definition, validation design, and reporting completeness. Many studies used the terms “combined”, “integrated”, or “fusion” without clearly specifying whether integration occurred at feature level, decision level, model level, or through a hybrid intermediate representation.

The review therefore found that the apparent superiority of fusion models should be interpreted cautiously. Reported performance gains may reflect not only fusion strategy, but also differences in dataset size, feature dimensionality, classifier selection, validation design, and study population. Because of this heterogeneity, formal meta-analysis was not considered appropriate. Instead, this review used structured subgroup synthesis across radiomics-only, deep learning-only, and fusion-based models to identify methodological patterns, limitations, and requirements for generalisable CDSS development.

To address these limitations, this review proposed a methodological framework that guides future DL–radiomics fusion studies through clearly defined clinical tasks, standardised imaging and segmentation protocols, reproducible radiomics and deep feature extraction, explicit fusion taxonomy, multicentre or site-held-out external validation, and transparent reporting of performance, calibration, explainability, and limitations. The proposed methodological framework is intended to support reproducible, interpretable, and generalisable clinical decision support for early lung cancer detection. Adherence to this framework, alongside decision-curve analysis, reader studies, prospective workflow evaluation, domain harmonisation, and fairness assessment, is necessary to move DL–radiomics fusion CDSS beyond retrospective proof-of-concept performance toward safe, interpretable, and generalisable clinical translation.

## Data Availability

Yes. This systematic literature review used data extracted from published studies identified through database searching and screening (study characteristics, methods, and reported performance outcomes).
